# Hydrogen Oxidation Benefits Alphaproteobacterial Methanotrophs Under Severe Methane Limitation

**DOI:** 10.1111/1462-2920.70163

**Published:** 2025-08-03

**Authors:** Ida F. Peterse, Arjan Pol, Geert Cremers, Tom Berben, Theo A. van Alen, Huub J. M. Op den Camp, Annelies J. Veraart, Sebastian Lücker

**Affiliations:** ^1^ Department of Microbiology Radboud Institute for Biological and Environmental Sciences, Faculty of Science, Radboud University Nijmegen the Netherlands; ^2^ Department of Ecology Radboud Institute for Biological and Environmental Sciences, Faculty of Science, Radboud University Nijmegen the Netherlands

**Keywords:** alphaproteobacterial methanotrophs, chemostat cultivation, hydrogen oxidation, hydrogenases, methane oxidation, *Methylocystis bryophila*

## Abstract

Hydrogen (H_2_) and methane (CH_4_) are produced in the anoxic layers of wetlands and sediments. In the overlaying oxygenated surface layers, these gases become available for oxidation by aerobic hydrogenotrophic and methanotrophic microorganisms. While H_2_ oxidation by verrucomicrobial methane‐oxidising bacteria (MOB) is extensively studied, less is known about this metabolism in MOB from the class *Alphaproteobacteria*, which frequently inhabit wetlands. We show that *Methylocystis bryophila* H2s^T^, *Methylocapsa aurea* KYG^T^, and “*Methylosinus acidophilus*” 29 encode diverse hydrogenases, instantly oxidise H_2_ when cultivated under CH_4_‐limited and low‐oxygen conditions, under which hydrogenase transcription is upregulated compared to CH_4_‐replete conditions. H_2_ exposure accelerated the maximum H_2_ oxidation rates but caused no upregulation of hydrogenases. Furthermore, while CH_4_ oxidation activity was affected by substrate‐limited growth conditions, H_2_ oxidation rates remained unaffected, and H_2_ supply to CH_4_‐limited chemostats caused increased biomass yield. Moreover, CH_4_ oxidation was severely inhibited by sulfide (H_2_S), while H_2_ and methanol oxidation rates were only moderately affected. In summary, the ability to conserve energy from H_2_ oxidation increases resilience and enhances growth of alphaproteobacterial methanotrophs in CH_4_‐limited environments, which revises the ecological role of these MOB in ecosystems with naturally fluctuating CH_4_ and H_2_ concentrations.

## Graphical abstract

We cultivated alphaproteobacterial methanotrophs under severe CH_4_‐limited conditions and studied their H_2_ oxidation characteristics as an alternative energy source.

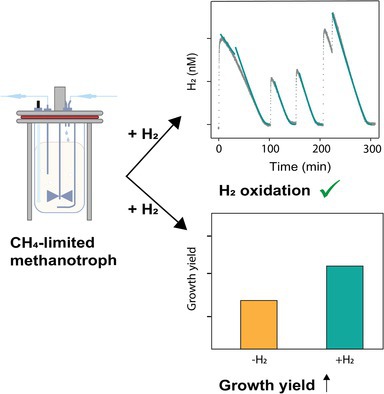

## 1 Introduction

Methane‐oxidising bacteria (MOB) play a crucial role in the mitigation of methane (CH_4_) emissions from ecosystems like wetlands, one of the largest natural sources of this potent greenhouse gas (Rosentreter et al. 2021; Saunois et al. 2019; Tian et al. 2016). In addition to CH_4_ oxidation, various aerobic MOB are capable of hydrogen (H_2_) oxidation (Carere et al. 2017; Hakobyan et al. 2020; Mohammadi et al. 2017; Mohammadi et al. 2019; Schmider et al. 2024; Schmitz et al. 2020). This metabolic flexibility may give MOB a competitive advantage in habitats with steep gradients of H_2_, CH_4_, and oxygen. Such gradients are characteristic of wetland soils and sediments, where organic matter degradation rapidly consumes available oxygen at the sediment surface. In the deeper layers, the organic material drives anaerobic processes, including sulfate reduction, fermentation, and methanogenesis, leading to the accumulation of H_2_, CH_4_, and sulfide (H_2_S). The H_2_ produced by fermentation serves as a substrate for anaerobic microorganisms like methanogenic archaea (Thauer et al. 2008), sulfate reducers (Muyzer and Stams 2008), and acetogens (Ragsdale and Pierce 2008). However, H_2_—along with CH_4_ and H_2_S—also diffuses upward into oxygenated surface layers, where it can potentially be aerobically oxidised by hydrogenase‐containing microbes, including several MOB (Greening et al. 2016; Piché‐Choquette and Constant 2019).

Typical aerobic MOB found in wetlands are representatives of the alphaproteobacterial genera *Methylocapsa*, *Methylosinus* and *Methylocystis* (Dedysh 2009; Dunfield et al. 2010; Jensen et al. 2023). Along with their gammaproteobacterial counterparts, these bacteria act as biological CH_4_ filters, reducing methane emissions to the atmosphere. While a continuous production of CH_4_ is expected in wetlands, its magnitude will largely be influenced by seasonal changes, water level dynamics and oxygen and substrate availability (Elberling et al. 2011). Low CH_4_ availability for MOB can occur depending on these factors and their position in the vertical CH_4_ gradient present in wetlands (Chasar et al. 2000; Strack and Waddington 2008). Alphaproteobacterial methanotrophs have been suggested to thrive under conditions with varying CH_4_ availability, have a more versatile substrate range than gammaproteobacterial MOB and are found to be stress tolerators (as reviewed by Ho et al. 2013). However, research on the CH_4_ and H_2_ oxidation capacity and H_2_S toxicity characteristics of alphaproteobacterial MOB under substrate limitation and low oxygen conditions is lacking. Such studies are expected to yield insights into the ecological relevance of MOB in dynamic and fluctuating habitats.

Recent studies have shown that verrucomicrobial MOB, such as *Methylacidiphilum fumariolicum* SolV and *Methylacidimicrobium tartarophylax* 4AC, can grow as true “Knallgas” bacteria, relying solely on H_2_, O_2_, and CO_2_. This hydrogenotrophic growth is facilitated by respiratory [NiFe] hydrogenases and the RuBisCO‐dependent Calvin‐Benson‐Bessham cycle for carbon fixation encoded in their genomes (Mohammadi et al. 2017; Mohammadi et al. 2019; Schmitz et al. 2020). In contrast, to date, no proteobacterial methanotrophs, which represent the majority of aerobic MOB, have been described to grow as autotrophs in the absence of CH_4_, as they typically are dependent on CH_4_ oxidation intermediates for carbon assimilation via either the serine cycle or RuMP pathway (Chistoserdova et al. 2009; Hanson and Hanson 1996). Yet, some of these methanotrophs, such as the gammaproteobacterial *Methylococcus capsulates* Strain Bath, do possess an active RubisCO enzyme and are dependent on CO_2_ in addition to CH_4_ (Henard et al. 2021). Strikingly, the alphaproteobacterial MOB *Methylocystis* sp. SC2 utilised H_2_ during growth on H_2_ and CH_4_, leading to a significantly increased biomass yield (Hakobyan et al. 2020). This suggests that alphaproteobacterial MOB possessing [NiFe] hydrogenases benefit from H_2_ oxidation.

In this study, we investigated the H_2_ oxidation capacities of three alphaproteobacterial methanotrophs grown under CH_4_ limitation and low oxygen saturation: *Methylocystis bryophila* H2s^T^, “*Methylosinus acidophilus*” strain 29, and *Methylocapsa aurea* KYG^T^. The first two were isolated from submerged acidic *Sphagnum* peat‐bog lakes in Germany (Belova et al. 2011; Belova et al. 2013) and the Netherlands (Raghoebarsing 2006), respectively, and the third from the sediment of an ephemeral stream (Dunfield et al. 2010). First, we examined the genomes of all three strains for [NiFe] hydrogenases. Subsequently, we tested their ability to oxidise H_2_ when grown under CH_4_‐limited conditions (as low as 80 ppm CH_4_) and low O_2_ (1%), and investigated their CH_4_ and H_2_ uptake kinetics. Moreover, we determined how the CH_4_ and H_2_ oxidation capacities of *M. bryophila* H2s^T^ changed under increasing CH_4_ limitation in the presence and absence of H_2_ and supported our findings with transcriptome analysis. Lastly, to deepen our insight into the stress tolerance of these versatile methanotrophs under CH_4_‐limited, H_2_‐replete conditions, we tested the toxicity of H_2_S on both CH_4_ and H_2_ oxidation (Table S1).

## 2 Materials and Methods

### 2.1 Species and Cultivation

The alphaproteobacterial methanotrophic species *Methylocystis bryophila* H2s^T^ (DSM 21852), *Methylocapsa aurea* KYG^T^ (DSM 22158), and “*Methylosinus acidophilus*” strain 29 (DSM 17628) were obtained from DSMZ (Braunschweig, Germany). They were grown in batch using 120 mL serum bottles capped with grey butyl rubber stoppers containing 20 mL species‐specific growth medium and 100 mL headspace consisting of 20% (v/v) CH_4_ in air. The bottles were incubated at 25°C–27°C on a shaking incubator at 200 rpm. The growth medium for *M. bryophila* H2s^T^ and “*M. acidophilus*” 29 was composed of 5 mM KNO_3_, 0.7 mM KH_2_PO_4_, 0.2 mM MgSO_4_·7 H_2_O, 7 μM CaCl_2_·2 H_2_O, and 0.3 mM NaCl. The final trace element concentration in the medium was 17 μM EDTA, 0.4 μM CuCl_2_, 7 μM FeSO_4_, 0.3 μM ZnSO_4_, 0.1 μM NiCl_2_, 1 μM CoCl_2_, and 0.1 μM Na_2_MoO_4_. The cultivation medium of *M. aurea* KYG^T^ consisted of 4.7 mM NaNO_3_, 0.9 mM MgSO_4_·6 H_2_O, 0.3 mM CaCl_2_·2 H_2_O, and trace elements as described earlier. In addition, 0.3% (v/v) of 5.5 mM iron(III)‐NTA solution and 0.1% (v/v) of 7.5 mM K_2_HPO_4_·3 H_2_O, 92.4 mM KH_2_PO_4_ solution were added to the medium.

### 2.2 Chemostat Cultivation

The three methanotrophic strains were cultivated in CH_4_‐limited chemostat bioreactors using the growth media described above. *M. bryophila* H2s^T^ was cultivated in a 1.5 L vessel controlled by an Applikon my‐Control system with the in‐Control software (Applikon, Delft, the Netherlands). The temperature in the liquid culture was 26°C–28°C without an external heating source as the motorised stirring at 1000 rpm produced heat. The vigorous stirring resulted in a mass transfer of CH_4_ gas to the liquid phase of at least 95%. The pH was maintained between 6 and 6.3 by automated addition of 0.5 M HCl. The concentration of dissolved oxygen (DO) in the culture was measured with an oxygen probe (Applikon AppliSense, Delft, the Netherlands) and automatically regulated at 5% air saturation by the supply of oxygen gas. During the initial set‐up, the chemostat received a gas flow of 10 mL min^−1^ Ar/CO_2_ (95:5% v/v). Starting at a culture dilution rate (D) of 0.006 h^−1^, the inlet CH_4_ concentration was about 2%, resulting in an optical density at 600 nm (OD_600_) of about 1. To reduce the growth rate of the culture, the dilution rate was first lowered to D = 0.003 h^−1^ and later to D = 0.0015 h^−1^ over a 2‐year period. Along with the dilution rates, the CH_4_ supply was reduced to maintain OD_600_ values between 0.6 and 1.1. CO_2_ was no longer supplied at these dilution rates, which had no effect on OD_600_. H_2_ gas was added to the gas mixture of the culture running at D = 0.0015 h^−1^ as a premixed gas of H_2_ and Ar (with a variable ratio depending on the experimental phase) at about 1 mL min^−1^. All gases were regulated by mass flow controllers (MFCs) and passed through a 0.22 μm filter to prevent contamination. Liquid was automatically pumped out of the chemostat when a level of 1.5 L was reached. “*M. acidophilus*” 29 was grown in a similar reactor set‐up as *M. bryophila* H2s^T^, at a constant dilution rate of D = 0.0057 h^−1^ but without H_2_ addition.

As the *M. bryophila* H2s^T^ culture encountered limitations upon long‐term H_2_ supply, the trace elements in the medium were changed to the following final concentrations: 2 μM CuCl_2_, 10 μM FeSO_4_, 1 μM ZnSO_4_, 1 μM NiCl_2_, 2 μM CoCl_2_, 1 μM MoO_4_Na_2_, 2 μM MnCl_2_, and 25 μM NTA. During CH_4_‐replete conditions with 1% and 10% O_2_, the trace elements were again slightly adapted, with the following final concentrations: 6 μM CuSO_4_, 4 μM FeSO_4_, 0.2 μM ZnSO_4_, 2 μM NiCl_2_, 0.2 μM CoCl_2_, 0.2 μM MoO_4_Na_2_, 4 μM MnCl_2_, 0.2 μM CeCl_3_, and 20 μM NTA.

*M. aurea* KYG^T^ was grown as continuous culture in a 3 L bioreactor (liquid volume 2.1 L) controlled by an ADI1010 controller unit (Applikon, Delft, the Netherlands). The temperature was regulated at 28°C using a water jacket. The pH was maintained between 6.05 and 6.15 by automatic addition of 0.5 M HCl. Initially, the chemostat received medium at a flow rate of 4 mL h^−1^ (D = 0.002 h^−1^), which was gradually changed to 2 mL h^−1^. Medium was pumped out of the chemostat when the volume exceeded 2.1 L. Using MFCs, the bioreactor was supplied with 0.12 mL min^−1^ CH_4_ and 7.5 mL min^−1^ Ar/CO_2_ (95:5% v/v) through a sterile 0.22 μm filter. The DO was 4%–5% air saturation and automatically regulated by the supply of O_2_ via a sparger. After this initial phase, methane supply to the bioreactor was improved as described in detail in the Supporting Information Material and Methods.

For all bioreactors, steady states were assumed after 4–5 reactor volume changes with constant OD_600_ and CH_4_ conversion rates. For assessing biomass yields, the concentration of gases in the inlet and outlet was accurately determined by gas chromatography and gas flow rates were measured by bubble flowmeters. CH_4_ was measured with a 7890B GC (Agilent Technologies, Santa Clara, USA) equipped with a flame ionisation detector set at 200°C with a Porapak Q column at 70°C. H_2_ and CO_2_ were detected using a HP 5890 gas chromatograph (Agilent, Santa Clara, USA) with a Porapak Q column at 30°C (1.8 m, ID 2 mm) and equipped with a thermal conductivity detector set at 140°C with argon as carrier gas.

### 2.3 H_2_ Oxidation Assays

Activity assays were performed using batch incubations in 120 mL glass serum bottles, capped with red butyl rubber stoppers (Rubber BV, the Netherlands). To prevent potential methanotroph inhibition, the stoppers were cleansed by boiling them three times in 100 mM NaOH before their first use. The capped bottles were made anoxic by evacuation and subsequent flushing with Ar, after which (v/v) 1%–20% O_2_, 0.5% CO_2_, and 0.5% H_2_ were added to the headspace. The chemostat cultures were diluted with medium to a final volume of 10 mL to prevent high hydrogen oxidation rates. Therefore, 5 mL of the *M. aurea* KYG^T^ culture (D = 0.002 h^−1^; OD_600_ = 0.54) and 2.5 mL of the “*M. acidophilus*” 29 culture (D = 0.0057 h^−1^; OD_600_ = 0.40) were diluted with 5 and 7.5 mL of medium, respectively. The *M. bryophila* H2s^T^ culture (D = 0.0016 h^−1^; OD_600_ = 0.53) was incubated undiluted. The bottles were incubated at 28°C on a gyratory shaker at 400 rpm for up to 3 days. For each measurement, H_2_ was detected as described above by injecting 100 μL headspace gas into the gas chromatograph multiple times per day.

### 2.4 Membrane‐Inlet Mass Spectrometry (MIMS)

Concentrations of dissolved H_2_ (*m*/*z* = 2) and CH_4_ (detected at *m*/*z* = 15 to limit co‐detection of other gases) were measured using MIMS (Hiden Analytical, Warrington, UK) as described by Schmitz et al. (2020, 2023) with slight modifications. For accurate kinetics of hydrogen consumption, a probe with a larger silicon membrane surface was used to achieve a lower detection limit. To avoid considerable consumption of the dissolved gases, we used a water‐jacketed 527 mL glass vessel instead of a 30 mL chamber (Figure S1). The probe was mounted through the stainless‐steel head plate clamped to the vessel with a Viton O‐ring. The probe was connected to the mass spectrometer via a water trap that consisted of a ¼ inch stainless steel tube, coiled three times with a coil diameter of 10 cm in a Dewar flask cooled to −80°C with solid CO_2_. Swagelok tube fitting unions with Vespel/Graphite ferrules were used for tubing connections. When the probe was connected to the vacuum inlet, the mass spectrometer operated for several days to reach stable low background signals.

Pieces of 1/16‐in. capillary PEEK tubes were fitted through multiple ports in the head plate with Omni‐Lok inverted cone fittings. One port was used for an optical oxygen probe (4 mm diameter; DP‐PST3, PreSens—Precision Sensing, Regensburg, Germany), sealed with an O‐ring, and connected to a Fibox 4 trace meter (PreSens). A magnetic stirrer bar was rotating at 1000 rpm on the bottom of the chamber. The chamber was filled with medium, retaining a small gas headspace of about 10–20 mL. This headspace was created during equilibration of the medium with Ar/CO_2_ (99:1% v/v). The gas was sparged at 40 mL min^−1^ for at least 30 min through a stainless‐steel capillary tube (0.7 mm outer diameter) inserted through one of the capillary ports. The central port served as the gas outlet. When the desired DO (ca. 2% air saturation) was reached, the steel capillary used for flushing was removed, and a 20 mL medium‐filled syringe was connected to the PEEK capillary of this port to fill up the chamber completely with medium while pushing out the gas headspace via the central port. The central capillary was then used to add culture, medium, or gases (CH_4_, H_2_, or O_2_, either pure or dissolved in water). These additions were done with gas‐tight Hamilton syringes (3‐in. needles, 0.74 mm diameter). Air‐saturated medium or pure O_2_ gas was added regularly to maintain the desired DO concentration during the respiration experiments. Pure O_2_ was used when higher O_2_ concentrations were required. The medium used during MIMS experiments was the same as for cultivation but using a concentration of 1 mM KNO_3_ to resemble conditions in the chemostat. Furthermore, the initial pH of the medium was increased to 6.4–6.8, and after flushing with Ar/CO_2_ gas, the pH of the medium was similar to the chemostat (6.0–6.2 ± 0.1 pH unit).

The apparent affinity (*K*_m(app)_) and maximum oxidation rate (*V*_max_) kinetic constants were determined by fitting a Michaelis–Menten curve through the CH_4_ and H_2_ consumption data. To allow for more accurate determination of the kinetic constants, H_2_ was replaced by D_2_ gas (*m*/*z* = 4) during some MIMS measurements because D_2_ signals were more stable.

### 2.5 Dry Weight Determination

Biomass dry weight (DW) was determined for the methanotrophic cultures at high and low growth rates. Culture suspensions (80 mL at OD_600_ = 1) were centrifuged at 15,000 × *g* for 20 min and dried to constant weight in a vacuum oven at 75°C. An OD_600_ = 1 of *M. bryophila* H2s^T^ culture growing at a dilution rate of 0.0016 h^−1^ corresponded to 295.8 ± 4.7 mg DW L^−1^ (*n* = 4). With higher dilution rates (D = 0.006 h^−1^ and 0.034 h^−1^), OD_600_ = 1 corresponded to 247.5 ± 4.6 mg DW L^−1^ (*n* = 4) and 252.3 ± 5.7 mg DW L^−1^ (*n* = 3), respectively. No DW was determined for D = 0.003 h^−1^. Instead, we used the average DW of the dilution rates 0.0016 and 0.006 h^−1^ (271.6 mg DW L^−1^) for calculating and comparing *V*_max_.

For *M. aurea* KYG^T^ (D = 0.002 h^−1^), OD_600_ = 1 was equivalent to 205.8 ± 5.6 mg DW L^−1^ (*n* = 4). Dry weight was only determined for the culture receiving both CH_4_ and H_2_. Determining dry weight for *M. bryophila* H2s^T^ under both conditions showed no difference, so we assumed the same for *M. aurea* KYG^T^. For “*M. acidophilus*” 29, no dry weight determinations were performed; however, we assumed that the dry weight would not differ significantly between the three strains used in this study, as their cell sizes are comparable (Belova et al. 2011; Dunfield et al. 2010; Raghoebarsing 2006). Therefore, we used the average DW of the other strains (250.3 mg DW L^−1^) for “*M. acidophilus*” 29.

### 2.6 DNA Extraction and Sequencing

DNA from a pure “*M. acidophilus*” 29 (DSM 17628) batch culture was extracted using CTAB extraction (Zhou et al. 1996) and sequenced with Nanopore MinION (Oxford Nanopore Technologies, Oxford, UK) and Illumina MiSeq (Illumina, San Diego, USA) technologies. Extracted DNA was quantified using Qubit dsDNA HS Assay Kit (Thermo Fisher Scientific Inc., Waltham, USA). To prepare the genomic DNA (350 ng total DNA) for Nanopore sequencing, the NEBNext FFPE DNA Repair Mix and NEBNext Ultra II End Repair/dA‐Tailing Module (New England Biolabs, Ipswich, MA, USA) were used according to the manufacturer's instructions. Library construction was performed using the Ligation Sequencing Kit 1D (SQK‐LSK109) and the Native barcodes (Native Barcoding Expansion Kit; EXP‐NBD104) following the manufacturer's instructions (Oxford Nanopore Technologies, Oxford, UK). Finally, the DNA library was loaded on a Flow Cell (R9.4.1) and sequenced using the MinION Mk1c device (Oxford Nanopore Technologies, Oxford, UK), according to the manufacturer's instructions.

The Illumina sequencing library was prepared using the Nextera XT Library Prep Kit (Illumina, San Diego, California, USA). Enzymatic tagmentation was performed with 1 ng of total DNA, indexed adapters were incorporated, and the libraries were amplified according to the manufacturer's instructions. After purification using AMPure XP beads (Beckman Coulter, Indianapolis, USA), the amplified library was checked for quality and size distribution with an Agilent 2100 Bioanalyzer using the High Sensitivity DNA kit (Agilent, Santa Clara, USA), and DNA quantity was checked with Qubit as described above. The library was paired‐end sequenced (2 × 300 bp) with the Illumina MiSeq machine using the MiSeq Reagent Kit (version 3, Illumina).

Furthermore, to assure the purity of the chemostat cultures “*M. acidophilus*” 29, *M. bryophila* H2s^T^, and *M. aurea* KYG^T^, DNA was sequenced using Illumina MiSeq technology as described above, using DNA isolated with the DNeasy Blood & Tissue Kit (Qiagen, Valencia, USA) following the manufacturer's instructions.

### 2.7 Genome Assemblies

The quality of the Illumina and Nanopore raw reads of the “*M. acidophilus*” 29 batch culture was assessed, and the raw Illumina reads were trimmed using CLC Genomics Workbench v22.0 (Qiagen Aarhus A/S, Denmark). Raw Nanopore reads were de novo assembled with NECAT (Chen et al. 2021), resulting in six contigs (see Table S2 for an overview of sequencing statistics). Next, these contigs were polished by mapping the Illumina reads against them using CLC Genomics Workbench. This resulted in a consensus sequence of the six contigs, which were annotated using Prokka (Seemann 2014).

Illumina sequencing reads of the three chemostat cultures were taxonomically classified with Kaiju (Menzel et al. 2016) to assess the purity of the sequenced sample. As the *Methylocystis* and *Methylosinus* cultures yielded reads of multiple species, further metagenomic analysis was performed (see Table S2 for an overview). Read quality was assessed with *FastQC* (Andrews 2010). Sequencing adapters were removed, and the reads were quality‐trimmed using the BBtools function BBduk (Bushnell 2014). The quality‐filtered, trimmed reads were assembled using SPAdes (Bankevich et al. 2012), and contig coverage was calculated with BBmap (Bushnell 2014). For the *Methylosinus* culture metagenome, genes were predicted on the assembled contigs using Prodigal (Hyatt et al. 2010). Predicted proteins were blasted against a curated [NiFe]‐hydrogenase database to detect all hydrogenases, including the ones belonging to contaminants. The average metagenomic read coverage of assembled contigs was calculated with coverM v0.7.0 (Aroney et al. 2025).

For the *Methylocystis* sample, contigs were binned into metagenome‐assembled genomes (MAGs) using CONCOCT (Alneberg et al. 2014), MaxBin2 (Wu et al. 2016), and MetaBAT 2 (Kang et al. 2019), and bins were dereplicated with DAS Tool (Sieber et al. 2018) using default parameter settings. Average MAG abundances in the metagenome were estimated with CheckM (Parks et al. 2015), MAG completeness and contamination with CheckM2 (Chklovski et al. 2023). The MAGs were classified taxonomically with GTDB‐Tk v2 (Chaumeil et al. 2022) and functionally annotated using Metascan (Cremers et al. 2022).

### 2.8 Hydrogenase Classification

The available genome sequences of *M. bryophila* s285 (GCF_002117405.1), which is identical to the *M. bryophila* H2s^T^ genome (GCF_027925445.1; 100% average nucleotide identity), and *M. aurea* KYG^T^ (GCF_000746085.1) were downloaded via GenBank and used for genome analysis focused on hydrogen metabolism. Hydrogenases were classified using the HydDB database (Søndergaard et al. 2016) and according to the presence of the binding motifs for [NiFe] and [FeS] centres in the large and small subunits, as described in Greening et al. (2016).

### 2.9 Phylogenetic Analysis

Sequences for heme‐copper oxidases (HCO) in *Methylocystis bryophila* H2s^T^ were initially identified by blastp searches. Previously classified anchor sequences were taken from the HCO subunit 1 tree by Bossis et al. (2014). Additional sequences necessary for improving bootstrap confidence values were collected using blastp searches against the nr database. Multiple alignment was performed using MUSCLE 3.8.1551 (Edgar 2004) and trimAl v1.4.rev22 (Capella‐Gutiérrez et al. 2009) was used to remove low‐scoring regions from the alignment (using the –automated1 flag). ModelTest‐NG v0.1.7 (Darriba et al. 2020) was used to determine the best‐scoring amino acid substitution model, which was LG + I + G4 + F (Le and Gascuel 2008) according to all three information criteria (BIC, AIC, and AICc). The maximum likelihood tree was generated using RAxML‐NG v1.2.2 (Kozlov et al. 2019) with an additional ML search of 100 random trees and 100 parsimony trees. Bootstrapping was performed with the autoMRE stopping criterion (Pattengale et al. 2010), which halted after 256 replicates. The resulting tree was visualised using FigTree 1.4.4 and finally marked up in Adobe Illustrator.

### 2.10 RNA Extraction and Sequencing

Triplicate biomass samples (equivalent to 25–30 mL of OD_600_ = 1) from steady‐state bioreactors of *M. bryophila* H2s^T^ and *M. aurea* KYG^T^ were centrifuged at 5000 × *g* for 15 min at 5°C, snap‐frozen with liquid nitrogen, and stored at −70°C. RNA was extracted using the RiboPure RNA Purification kit (Ambion, Thermo Fisher Scientific Inc., Waltham, USA) according to the manufacturer's instructions, with the addition of a 1 min bead‐beating step at full speed using a Tissuelyser LT (Qiagen). The extracted RNA was cleaned with DNAse I (Thermo Fisher Scientific) for 60 min at 37°C, after which the total RNA was purified and concentrated with the Zymo RNA Clean & Concentrator‐5 kit (Zymo Research, Irvine, California, USA).

cDNA libraries were built with the Stranded Total RNA Prep Ligation with Ribo‐Zero Plus kit (Illumina) which included a rRNA depletion step. Library quantitation with Qubit and cDNA library quality on the Bioanalyzer were assessed as described above. Libraries were paired‐end sequenced (150 bp) on the NovaSeq X system (Macrogen Inc., Amsterdam, the Netherlands).

Read quality was assessed with *FastQC* (Andrews 2010). Sequencing adapter removal and read quality trimming were done using the BBtools function BBduk (Bushnell 2014) with the following settings: k = 23, mink = 11, hdist = 1, ktrim = *r*, tpe, tbo, qtrim = rl, trimq = 18, maq = 24, maxns = 0, ftm = 5 and minlen = 60 (see Table S2 for an overview of sequencing statistics). Reads were mapped against the reference genomes of *M. aurea* KYG^T^ and *M. bryophila* H2s^T^ obtained from Genbank as described in the previous section using *Kallisto* version 0.50.1 (Bray et al. 2016). After mapping, a total of 55 and 57 hits to rRNA, tRNA, and ncRNA regions were removed from the datasets from *M. aurea* KYG^T^ and *M. bryophila* H2s^T^, respectively. Mapped read counts were converted to transcripts per million (TPM) to compare expression levels of genes within a sample, between replicates and across varying conditions. Significant statistical differences were detected using the *voom* function of the R package limma (Law et al. 2014; Smyth 2005). Log_2_‐fold changes ≥ 1 and with an adjusted *p* < 0.01 were considered significant.

## 3 Results

### 3.1 Genomic Potential for H_2_ Oxidation of *M. bryophila* H2s^T^ , *M. aurea* KYG^T^ , and “*M. acidophilus*” 29

**FIGURE 1:**
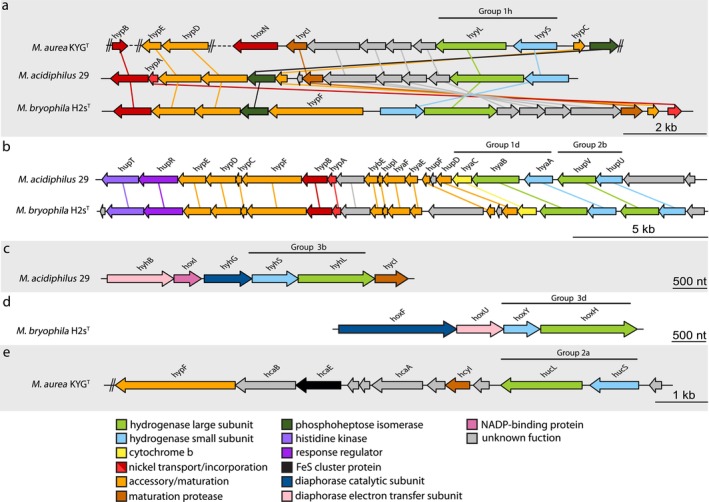
Gene arrangement of all [NiFe] hydrogenases in the genomes of the three alphaproteobacterial methanotrophs *M. aurea* KYG^T^, “*M. acidophilus*” 29, and *M. bryophila* H2s^T^. (a) Group 1h uptake hydrogenases (Hyd‐1h) were present in all three genomes; (b) group 1d uptake hydrogenases (Hyd‐1d) and group 2b regulatory hydrogenases (Hyd‐2b) were found in one gene cluster in both “*M. acidophilus*” 29 and *M. bryophila* H2s^T^. “*M. acidophilus*” 29 and *M. bryophila* H2s^T^ genomes encoded for (c) group 3b (Hyd‐3b) and (d) group 3d (Hyd‐3d) bidirectional hydrogenases. (e) The group 2a uptake hydrogenase (Hyd‐2a) encoded in the *M. aurea* KYG^T^ genome. The arrows represent genes, indicate the direction of transcription, and are drawn to scale. Homologues are connected by lines. Double lines indicate breaks in the genomic assembly and dashed lines indicate a non‐scaled genomic region.

The genomes of the three studied methanotrophs all contain multiple gene clusters encoding [NiFe] hydrogenases (Figure 1; Table S3), indicating the metabolic potential for H_2_ oxidation. All three organisms possess a group 1h [NiFe] H_2_‐uptake hydrogenase (Hyd‐1h) within a largely conserved gene cluster (Figure 1a). The encoded large subunits HhyL (msin_004647, B1812_08935, DL86_RS14135) contain the complete L1 and L2 motifs (see Greening et al. 2016 for hydrogenase motifs), along with the conserved cysteine residues for [NiFe] binding. The small subunit HhyS contains binding motifs for three [FeS] clusters and is encoded directly downstream of *hhyL*. The upstream region of *hhyL* includes four protein‐coding sequences (CDS) of unknown function. In the cases of *M. aurea* KYG^T^ and “*M. acidophilus*” 29, the CDS directly upstream of *hhyL* has a NifU‐like domain and may be involved in [FeS] cluster biosynthesis (Ayala‐Castro et al. 2008). This domain appears to be absent in the corresponding CDS in *M. bryophila* H2s^T^ (B1812_08940), which nonetheless shares up to 50% amino acid identity with other alphaproteobacterial NifU‐like proteins.

Furthermore, *M. bryophila* H2s^T^ and “*M. acidophilus*” 29 possess a group 1d [NiFe] H_2_‐uptake hydrogenase (Hyd‐1d; Figure 1b), which is considered an oxygen‐tolerant hydrogenase. However, in *Methylacidiphilum fumariolicum* SolV, Hyd‐1d was only active at dissolved O_2_ concentrations below 0.2% (Mohammadi et al. 2017; Schmitz et al. 2020). The Hyd‐1d‐encoding regions in the *M. bryophila* H2s^T^ (B1812_13015 to B1812_13130) and “*M. acidophilus*” 29 genomes (msin_03306 to msin_03328) also include genes for a H_2_‐sensing group 2b [NiFe] hydrogenase (Hyd‐2b; Figure 1b). The catalytic subunits of Hyd‐2b (HupU/HupV) are believed to interact with the regulatory histidine kinase (HupT) and transcriptional regulator (HupR) encoded upstream in the same gene cluster. A similar regulatory hydrogenase from *Cupriavidus necator* (formerly known as *Ralstonia eutropha* H16) has been extensively studied and shown to activate the transcription of membrane‐associated respiratory hydrogenases when sensing H_2_ (Bernhard et al. 2001; Buhrke et al. 2004; Buhrke et al. 2005). *M. bryophila* H2s^T^ and “*M. acidophilus*” 29 have a CDS region (B1812_13060 and msin_03314, respectively) adjacent to the Hyd‐2b genes, which share 32% and 36% protein identity with the large subunit HupV of *M. bryophila* H2s^T^ and “*M. acidophilus*” 29, respectively. Despite being classified as a Hyd‐2b by HydDB (Søndergaard et al. 2016), both motifs are incomplete and lack multiple cysteine residues essential for binding the catalytic [NiFe] cluster.

The genomes of *M. bryophila* H2s^T^ and “*M. acidophilus*” 29 also contain bidirectional [NiFe] hydrogenases (Figure 1c,d). These widely distributed Group 3 hydrogenases are oxygen‐tolerant enzymes and are believed to facilitate the reversible generation of NAD(P)H through H_2_ oxidation (Greening et al. 2016). This process generates reductive power for processes such as CH_4_ oxidation. “*M. acidophilus*” 29 encodes a group 3b [NiFe] hydrogenase (Hyd‐3b) featuring the motifs L1 and L2 in the large subunit (HyhL; msin_00436). The gene cluster for this Hyd‐3b also encodes a protein (HoxI) that possesses a cyclic nucleotide‐binding domain similar to the one found in the catabolite gene activator cAMP and is thought to bind NADP in addition to NAD^+^ (Burgdorf et al. 2005). Meanwhile, *M. bryophila* H2s^T^ encodes a group 3d [NiFe] hydrogenase (HoxH; B1812_19420) but lacks the *hoxI* gene.

Interestingly, *M. aurea* KYG^T^ possesses another high‐affinity hydrogenase in addition to Hyd‐1h, which is believed to scavenge atmospheric H_2_. This enzyme belongs to the group 2a H_2_‐uptake hydrogenases (Hyd‐2a; Figure 1e). The large subunit of Hyd‐2a (HucL; DL86_RS13145) is encoded in a gene cluster along with the small subunit (HucS) and a Rieske‐type FeS cluster protein (HucE). Additionally, the maturation protease HycI and the highly conserved maturation factor HypF are also encoded in this cluster (Hogendoorn et al. 2020; Islam et al. 2020).

Lastly, in addition to the hydrogenases' catalytic sites and FeS cluster proteins, the methanotrophs' genomes contain multiple Ni^2+^ transport and incorporation proteins, maturation proteases, and accessory proteins (Figure 1; Table S3). These proteins are often part of hydrogenase gene clusters, such as the *hypABFCDE* gene cluster upstream of Hyd‐2b and Hyd‐1d in “*M. acidophilus*” 29 and *M. bryophila* H2s^T^ (Figure 1b).

### 3.2 H_2_ Oxidation of Three Methanotrophic Species Grown on Limited CH_4_ and Low O_2_

**FIGURE 2:**
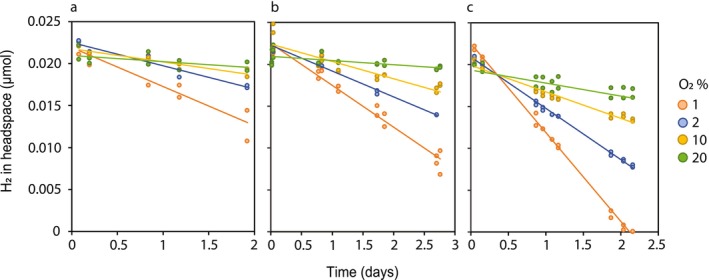
H_2_ oxidation by (a) *M. aurea* KYG^T^, (b) “*M. acidophilus*” 29, and (c) *M. bryophila* H2s^T^ under O_2_ concentrations ranging from 1% to 20% O_2_. Biomass originated from CH_4_ and O_2_‐limited chemostat cultures. Every dot represents the average of duplicate measurements, and a trendline was fitted through all data points obtained from replicate incubations (*n* = 2–4).

To demonstrate that the observed metabolic potential truly allows H_2_ utilisation in these three methanotrophs, their H_2_‐oxidising capacity was tested across a range of oxygen levels. To achieve this, all three strains were pre‐grown under CH_4_ limitation (see Text S1 for calculations) and low dissolved oxygen concentrations (1% O_2_). Upon transfer to bottles containing 5000 ppm H_2_ without any added CH_4_, all three cultures immediately began oxidising H_2_ (Figure 2). Maximal oxidation rates were recorded at the lowest O_2_ concentration (1% O_2_), and H_2_ oxidation was inhibited at O_2_ concentrations above 2%.

### 3.3 Maximum H_2_ Oxidation Rate Accelerates Over Time

**FIGURE 3:**
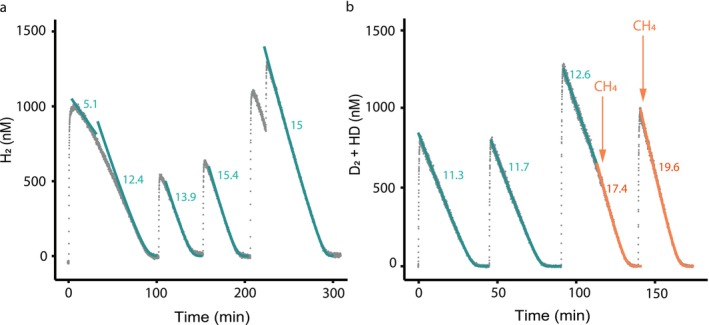
H_2_/D_2_ oxidation by *Methylocystis bryophila* H2s^T^. (a) Oxidation rates accelerate over repeated H_2_ pulses, and (b) further upon the addition of 1 μM CH_4_ (orange arrows at 125 and 150 min). The culture in (b) was pre‐incubated with H_2_ for 6 h before the addition of D_2_. The data points represent the sum of D_2_ and HD, as HD is formed through H^+^/D^+^ ion exchange between D_2_ and H_2_O. Kinetic parameters were determined by fitting simulated Michaelis–Menten kinetics through the data points. Rates are displayed in μmol min^−1^ g^−1^ DW. Additional data on the oxidation kinetics of all three alphaproteobacterial methanotrophs are provided in Table 1.

After confirming H_2_ oxidation, we closely monitored the oxidation of H_2_ and CH_4_ in *Methylocystis bryophila* H2s^T^ using a MIMS system, always at 1%–2% dissolved O_2_. This technique allowed us to determine Michaelis–Menten enzyme kinetics without mass transfer limitations between the gas and liquid phases, which can occur during batch incubations with a gas headspace. Without pre‐incubation on H_2_, the initial activity of *M. bryophila* H2s^T^ cells grown under CH_4_ limitation (0.44 μM CH_4_ at D = 0.006 h^−1^ as estimated in Text S1) was 5.1 μmol min^−1^ g^−1^ DW (Figure 3a). Subsequently, supplying additional H_2_ pulses (600 nM to 1 μM H_2_) over 3 h gradually increased the apparent *V*_max_ to 15.0 μmol min^−1^ g^−1^ DW (Figure 3a; Table 1). Comparably, the initial H_2_ oxidation rate was 12.0–14.7 μmol min^−1^ g^−1^ DW when the *M. bryophila* H2s^T^ culture had been pre‐incubated with H_2_ for 3–5 h in the MIMS cell. Maximum H_2_ oxidation rates of 20.8 μmol min^−1^ g^−1^ DW were recorded after incubating cells with H_2_ overnight (Table 1). In contrast, CH_4_ oxidation rates remained constant when CH_4_ was added frequently.

**TABLE 1:** Kinetic constants of CH_4_ and H_2_ oxidation by the three alphaproteobacterial methanotrophs growing in CH_4_ and O_2_‐limited chemostats at different growth rates.

Species	Growth rate (h^−1^)^a^	*V*_max_ CH_4_	*V*_max_ H_2_	*V*_max_ H_2_/*V*_max_ CH_4_ ^b^	*K*_m(app)_ CH_4_	*K*_m(app)_ H_2_
*Methylocystis bryophila* H2s^T^	0.065^c^	159	0.6 → 14	0.09	3.5	n.d.
	0.006	155	8 → 20^d^	0.13	3	0.03–0.06
	0.003	84	n.d.	n.d.	2.5–3	n.d.
	0.0016	61	4 → 24^d^	0.39	1–2	0.15–0.20
*“Methylosinus acidophilus”* 29	0.0057	75	11 → 72^d^	0.94	2.5–4	0.04–0.07
*Methylocapsa aurea* KYG^T^	0.002	93	4 → 30^d^	0.32	3.5	0.04–0.10

*Note:* The maximum H_2_ oxidation rate shows the range from the initial rate to the rate measured after prolonged H_2_ exposure. All rates are in μmol min^−1^ g^−1^ DW and apparent affinity constants (*K*_m(app)_) in μM. n.d., not determined.

^a^ The growth rate equals the dilution rate in continuous culture.

^b^ The ratio was calculated using the highest *V*_max_ measured for H_2_ oxidation.

^c^ Cultivated as batch culture instead of chemostat culture.

^d^ The arrow indicates the increase in H_2_ oxidation rate over time when cells are exposed to H_2_.

MIMS measurements were most accurate when D_2_ was added instead of H_2_, as the D_2_‐specific *m*/*z* of 4 has very low and stable background noise, in contrast to the *m*/*z* 2 peak, which also results from the fragmentation of residual H_2_O and CH_4_. For *M. bryophila* H2s^T^ cells pre‐grown under CH_4_‐limited conditions (D = 0.006 h^−1^), the apparent affinity constant (*K*_m(app)_) for H_2_/D_2_ was 35 ± 5 nM (*n* = 10; Figure 3). Upon the addition of CH_4_, we measured an immediate increase in the D_2_ consumption rate from 12.6 to 17.4 μmol min^−1^ g^−1^ DW, and the *K*_m(app)_ increased to 55 nM H_2_/D_2_ (Figure 3b).

As we anticipated that H_2_ consumption of the culture would be increasingly important as an additional energy source under severe CH_4_ limitation, we reduced the dilution rate of the CH_4_‐limited and low dissolved O_2_ (1%) *M. bryophila* H2s^T^ chemostat twice over a span of 2 years: first from D = 0.006 h^−1^ to D = 0.003 h^−1^, and then to D = 0.0016 h^−1^, with the latter operated at a dissolved CH_4_ concentration of 0.11 μM (see Text S1). This resulted in an almost threefold decrease in the maximum CH_4_ oxidation rate, which dropped from 155 to 61 μmol min^−1^ g^−1^ DW (Table 1). However, the *V*_max_ of H_2_ oxidation remained stable, leading to a reduced difference between the CH_4_ and H_2_ oxidation rates. Furthermore, the affinities for both CH_4_ and H_2_ were lower at these dilution rates (Table 1). Using the experimentally obtained kinetic parameters from biomass cultivated in a CH_4_‐limited chemostat, we simulated Michaelis–Menten kinetics for H_2_ and CH_4_ oxidation (Figure S2a). This simulation indicated that H_2_ oxidation rates can exceed CH_4_ oxidation rates when the CH_4_ concentration drops below 0.75 μM. This was also verified experimentally in the MIMS cell when both substrates were simultaneously added; H_2_ oxidation rates overtook CH_4_ oxidation rates below a substrate concentration of 0.5 μM (Figure S2b).

Finally, to determine the effect of CH_4_ limitation, we used MIMS to determine Michaelis–Menten kinetics for CH_4_ and H_2_ oxidation in *M. bryophila* H2s^T^, which was pre‐grown in batch culture without CH_4_ limitation (*μ* = 0.065 h^−1^). This resulted in a maximum CH_4_ oxidation rate of 159 μmol min^−1^ g^−1^ DW with a *K*_m(app)_ of 3.5 μM CH_4_ (Table 1). The maximum oxidation rate of H_2_ gradually increased with the addition of H_2_ pulses, rising from 0.64 to 14.2 μmol min^−1^ g^−1^ DW over 43 h (Figure S3), demonstrating that H_2_ oxidation also occurs under CH_4_‐replete conditions.

Similar experiments were conducted with “*M. acidophilus*” 29 and *M. aurea* KYG^T^ biomass cultivated in chemostat cultures. For these methanotrophic species, we determined the kinetic constants for a single growth rate only; these chemostats were maintained under stable dilution rates of D = 0.0057 h^−1^ for “*M. acidophilus*” 29 and D = 0.002 h^−1^ for *M. aurea* KYG^T^. The apparent kinetic constants were of the same order of magnitude as those determined for *M. bryophila* H2s^T^ (Table 1). Notably, the H_2_ oxidation rates of “*M. acidophilus*” 29 during MIMS incubations accelerated over time, even more so than those measured for the other two species, reaching rates comparable to those found for CH_4_ oxidation. However, sequencing the chemostat culture also revealed the presence of a *Burkholderia* contaminant possessing a Group 3d [NiFe] hydrogenase (Table S2). Thus, although “*M. acidophilus*” 29 was fourfold more abundant in the culture than the *Burkholderia* contaminant based on average metagenomic read coverage, we cannot conclude that H_2_ oxidation activity is solely attributable to “*M. acidophilus*” 29.

We also checked the metagenomes of the *M. bryophila* H2s^T^ and *M. aurea* KYG^T^ bioreactors for contaminants to verify that H_2_ oxidation activity is purely from the methanotrophs in the cultures. Using read‐based taxonomic classification, we identified the *M. aurea* KYG^T^ culture as free of contaminants (Figure S4). The metagenome of the *M. bryophila* H2s^T^ bioreactor contained a *Variovorax* contaminant representing 4% of the sequenced reads, but no hydrogenase‐encoding genes were found in the corresponding bin (see Text S2 and Table S2). Additionally, bioreactor biomass incubated in bottles with medium under H_2_ and CO_2_ did not show growth, thus excluding the presence of an autotrophic hydrogen consumer.

### 3.4 H_2_ Supply Increases Growth Yield Under CH_4_ Limitation

**FIGURE 4:**
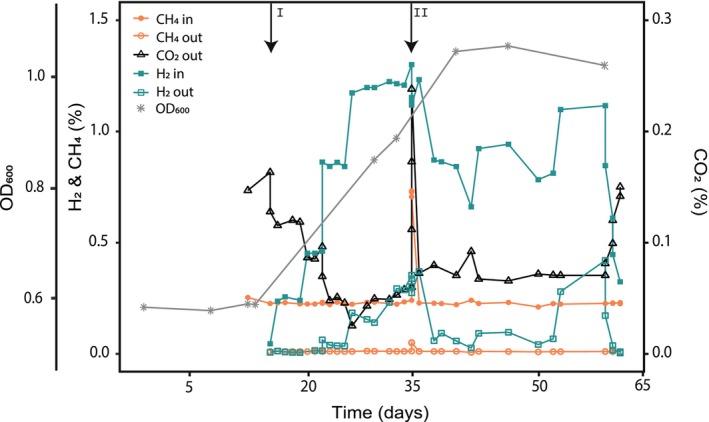
Long‐term supply of H_2_ gas to a CH_4_ and O_2_‐limited chemostat culture of *Methylocystis bryophila* H2s^T^. CH_4_ (circles) and H_2_ (squares) concentrations in the gas inflow (filled symbols) and CH_4_, H_2_, and CO_2_ (triangles) in the gas outflow (open symbols) of the bioreactor were followed. The H_2_ concentration in the gas inflow was increased and decreased periodically. The first arrow (I) indicates the start of H_2_ supply, the second one (II) the short‐term increase of CH_4_ supply.

To monitor the effect of H_2_ on growth yield, H_2_ was supplied to an *M. bryophila* H2s^T^ chemostat culture, with cells growing under growth‐limited conditions at D = 0.0016 h^−1^ with a stable CH_4_ supply (0.23% (v/v) in the gas inflow; Figure 4). During this time, H_2_ and CH_4_ concentrations in the gas inflow of the chemostat were monitored, along with H_2_, CH_4_, and CO_2_ concentrations in the outflow. All CO_2_ measured in the gas outflow was produced by the culture, as no additional CO_2_ was supplied to the chemostat during this period. Upon the addition of H_2_ to the inflow, CO_2_ production dropped (Figure 4, first arrow), indicating more CH_4_ oxidation intermediates were available for carbon assimilation. When the H_2_ supply to the chemostat was raised further, the H_2_ in the outflow increased, indicating the culture's inability to oxidise all H_2_ supplied. Contrastingly, the CO_2_ concentration in the outflow decreased while there was no change in CH_4_ concentration in the outflow. Correspondingly, the OD_600_ of the culture nearly doubled from 0.6 to 1.0 upon the addition of H_2_. A short‐term elevation of the CH_4_ supply (from 0.23% to 0.73%) resulted in a rapid fourfold increase of CO_2_ in the outflow (from 0.06% to 0.24%) but did not cause an immediate boost in H_2_ oxidation rates, indicating that the cells operated at maximal H_2_ oxidation capacity (Figure 4, second arrow).

Finally, when the H_2_ concentration in the inflow was decreased again, CO_2_ immediately increased in the outflow, indicating more CH_4_ oxidation was required for energy metabolism. Long‐term H_2_ supply caused an increase in H_2_ outflow, implying that the culture encountered limitations (Figure S5). The trace element composition was adapted from EDTA to NTA as a chelating agent with the additional supply of the metal cofactors Ni^2+^ and Cu^2+^, which are needed for the catalytic centre of hydrogenases. Indeed, after this medium modification, the OD_600_ of the culture increased further, and the H_2_ levels in the outflow dropped again (Figure S5). Under these stable H_2_‐replete conditions, the total H_2_ consumption of the culture exceeded the CH_4_ consumption by a factor of 6 to 7. The calculated yield of the culture without H_2_ compared to with H_2_ supply increased from 5.8 to 13.1 g DW mol^−1^ CH_4_, respectively. However, after H_2_ was again omitted from the gas supply, the newly calculated yield of *M. bryophila* H2s^T^ on CH_4_ alone was 7.5 g DW mol^−1^ CH_4_, likely because the culture was limited in trace elements during the first period of the experiment (Figure S5). Therefore, the effect of H_2_ on the growth yield of a purified *M. bryophila* H2s^T^ culture (D = 0.006 h^−1^) was reassessed using the adapted medium. When the bioreactor reached steady state and H_2_ was added, consumption started immediately and was 3‐fold higher than the CH_4_ consumption. During H_2_ supply, the conversion of CH_4_ to CO_2_ dropped from 55% ± 1% to 13% ± 0.4% (*n* = 5 days), indicating that carbon incorporation from CH_4_ into biomass increased from 45% to 87%. This also matched the observed doubling of biomass content (OD_600_ from 0.94 to 1.83), corresponding to a yield increase from 6.9 to 14 g DW mol^−1^ CH_4_, confirming earlier observations.

Furthermore, to verify the yield increase upon H_2_ addition also for *M. aurea* KYG^T^, a similar experiment was performed with the CH_4_‐limited bioreactor culture (D = 0.001 h^−1^). H_2_ consumption was instantaneous and 6‐ to 7‐fold higher than CH_4_ consumption. Furthermore, a drop of CO_2_ in the gas outflow of the bioreactor was measured after H_2_ supply (Table S4), and based on OD_600_ increase and CH_4_ consumption, we calculated a yield increase from 2.8 to 5.4 g DW mol CH_4_^−1^ (Table S4).

### 3.5 H_2_S Inhibition of CH_4_ , Methanol, and H_2_ Oxidation

**FIGURE 5:**
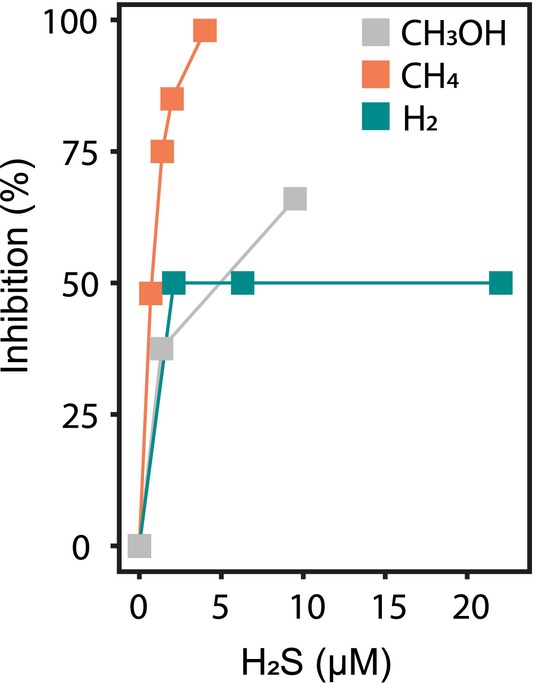
Inhibiting effect of H_2_S on the CH_4_, MeOH, and H_2_ oxidation rates by *Methylocystis bryophila* H2s^T^. CH_4_ and H_2_ oxidation rates were measured in the MIMS and CH_3_OH consumption (1.5 mM) was measured via O_2_ respiration rates (Figure S7).

Presence of sulfide: quinone reductase (SQR) genes in all three genomes (B1812_04910; DL86_RS09005; msin_02960) prompted us to test the effects of H_2_S toxicity on CH_4_, methanol (CH_3_OH; MeOH), and H_2_ oxidation by *M. bryophila* H2s^T^, as H_2_S gas often co‐occurs with CH_4_ and H_2_ in habitats experiencing (temporal) anoxia. When 0.7 μM, 1.4 μM, and 2 μM H_2_S were added to the MIMS cell containing the *M. bryophila* H2s^T^ culture (D = 0.003 h^−1^), the CH_4_ oxidation rate was inhibited by 48%, 74%, and 85%, respectively (Figure 5 and S6). A similar experiment was conducted using MeOH as the electron donor and carbon source instead of CH_4_. The MeOH respiration rate—measured as O_2_ consumption—was significantly less inhibited than the CH_4_ respiration rate, and only 66% inhibition was observed when 9.5 μM H_2_S was added to the cells (Figures 5 and S7).

Interestingly, with H_2_ as the only electron donor, additions of increasing H_2_S concentrations to a final concentration of 20 μM resulted in a 50% inhibition of H_2_ oxidation only, from 5 to 2.3 μmol min^−1^ g^−1^ DW. Because H_2_ oxidation activity was partly resistant to high H_2_S concentrations, a sulfide‐insensitive terminal oxidase is expected to be present. Therefore, a phylogenetic tree of heme copper oxidase (HCO) amino acid sequences was calculated (Figure S8). *M. bryophila* H2s^T^ possesses a *bo3*‐type (B1812_07680) and several *aa3*/*caa3*‐type (B1812_05015, B1812_18765 and B1812_21535) but no *ba3*‐type cytochrome *c* oxidase, which was discussed by Schmitz et al. (2023) to be a sulfide‐insensitive terminal oxidase of *M. fumariolicum* SolV. However, the evolutionary HCO‐unrelated *bd*‐cytochrome oxidase (B1812_10350) is encoded in the genome and was previously reported to be sulfide‐insensitive, too (Forte et al. 2016).

### 3.6 H_2_ Availability and CH_4_ Limitation Drive Hydrogenase Gene Expression

**FIGURE 6:**
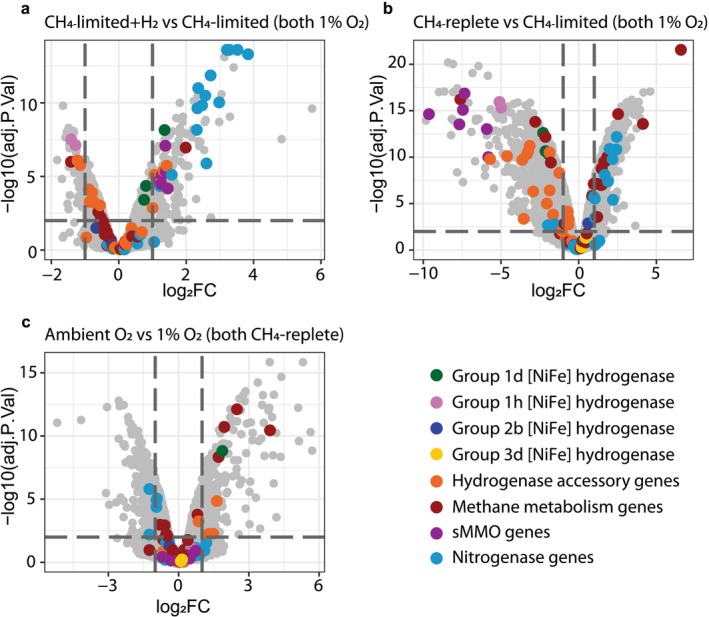
Volcano plots illustrating differential gene expression of *Methylocystis bryophila* H2s^T^. Shown is the log_2_‐fold change (log2FC) in transcription between cells cultivated (a) under CH_4_‐limited conditions with and without 1% H_2_ supplied, (b) between CH_4_‐replete and CH_4_‐limited cells, and (c) between fast‐growing cells cultivated at ambient and 1% O_2_. The *x*‐axis shows the log_2_‐fold change of transcript abundance in the first compared to the second condition. The *y*‐axis represents the negative log_10_ of the adjusted *p* value (−log10(adj.P.Val)), indicating the false discovery rate corrected for multiple testing. Significance thresholds, indicated by grey dashed lines, are set at a log_2_‐fold change ≥ 1 and an adjusted *p* value ≤ 0.01.

*M. bryophila* H2s^T^ biomass was cultivated under four different conditions to identify drivers of hydrogenase gene expression (Figure 6); CH_4_‐limited with and without H_2_ addition, and CH_4_‐replete with high O_2_ (20%) and low O_2_ (1%) concentrations. The transcriptomes of *M. bryophila* H2s^T^ grown under CH_4_ limitation with and without H_2_ supply clustered together, based on a principal component analysis (Figure S9a), and only 223 genes were differentially expressed (log_2_‐fold change (LFC) ≥ 1 and adjusted *p* value ≤ 0.01). The largest number of genes was differentially expressed when comparing the CH_4_‐replete and CH_4_‐limited conditions (1565 genes; Figure S9b). Moreover, gene expression of *M. aurea* KYG^T^ was investigated for two different conditions, CH_4_‐limited with and without H_2_ addition. In total, 231 genes were differentially expressed, of which 110 were upregulated and 121 were downregulated in the CH_4_‐limited condition compared to the condition with H_2_ (Figure S9c,d). Upon H_2_ supply to the chemostat, no significant differential gene expression of hydrogenases could be detected (Figure S10; Table S5), and no major trends could be detected in general carbon and nitrogen metabolism (see Text S3 for further description).

When grown under CH_4_ limitation, H_2_ addition to the *M. bryophila* H2s^T^ culture caused a downregulation of the Group 1h [NiFe] hydrogenase large and small subunit expression of LFC 1.3 and 1.4, respectively (Figure 6a). The addition of H_2_ furthermore induced a small but insignificant upregulation of the Group 1d hydrogenase small and large subunits (LFC 0.75 and 0.81), and a significant upregulation of the *b*‐type cytochrome subunit (LFC 1.4) and the accessory genes located upstream in the gene cluster (Figures 1 and 6a; Table S5).

In the absence of H_2_, lifting the CH_4_‐limitation led to a downregulation of both Group 1h hydrogenase subunits (LFC 5.0 and 5.1), the Group 1d subunits, including the *b*‐type cytochrome protein and most hydrogenase accessory genes (Figure 6b; Table S5). No differential gene expression for the Group 2b and Group 3d [NiFe] hydrogenases was found for any of the conditions, and their transcription level was low (TPM ≤ 50, compared to an average TPM of 235).

The addition of H_2_ to the *M. bryophila* H2s^T^ culture growing under CH_4_ limitation induced the significant upregulation of genes encoding the soluble methane monooxygenase (*mmoXYBZDC*; LFC 1.1–1.5) due to copper limitation, nitrogenase (*nifKDH*; LFC 3.3–3.8) and the Fix complex involved in shuttling electrons from NADH to the nitrogenase complex (*fixABC*; LFC 2.2–2.4; Figure 6a; Table S5). Moreover, *nifKDH* and *fixABC* were also upregulated (LFC 2.0–2.5 and 1.7–1.9, respectively) at CH_4_‐replete fast‐growing conditions, as were the genes encoding the three particulate methane monooxygenase operons (two almost identical copies of pMMO_1 and one pMMO_2). The two *pmoCAB_*1 operons of *M. bryophila* H2s^T^ (starting at B1812_14770 and B1812_18675; both LFC 1.5–1.7) are 100% identical, except for their *pmoC* (99.2% average nucleotide identity), allowing distinction of their expression levels (Table S5). In the distinct pMMO_2 operon, only *pmoAB*_2 (B1812_13465 and B1812_13470) showed significant upregulation (LFC 1.2) during the CH_4_‐replete condition. In addition, the lanthanide‐dependent methanol dehydrogenase was significantly upregulated during this condition (*xoxF*; LFC 6.6), which, however, likely was caused by an adaptation of the trace element solution, including the addition of lanthanides, during the CH_4_‐replete conditions. In contrast, the calcium‐dependent methanol dehydrogenase (MDH) complex (*mxaFJGIRSACK*; LFC 1–2.9) and all sMMO subunits were downregulated (LFC 5.9–9.6) compared to the CH_4_‐limited condition. (Figure 6b; Table S5).

Lastly, a pairwise comparison of fast‐growing *M. bryophila* H2s^T^ cells at the high and low O_2_ conditions showed upregulation of the whole Ca‐dependent MDH complex, a monocistronic *pmoC* subunit (B1812_06380; LFC 3.9), and the small subunit of the Group 1d [NiFe] hydrogenase cluster under ambient O_2_ conditions (Figure 6c; Table S5).

## 4 Discussion

In the past decade, (meta)genomic studies have shown that H_2_ oxidation is a widespread trait among microorganisms (Greening et al. 2016), challenging the notion that H_2_ metabolism is a niche trait exclusive to microbes found in ecosystems rich in H_2_, such as volcanic regions, animal guts, and anoxic sediments. For instance, hydrogenases have been detected in proteobacterial methanotrophic genomes (Hakobyan et al. 2020), and several studies indicate H_2_ oxidation activity in alphaproteobacterial methanotrophs (Hakobyan et al. 2020; Schmider et al. 2024). Although these methanotrophs often endure substrate limitation, understanding othe CH_4_ and H_2_ oxidation capacity of alphaproteobacterial MOB under such conditions is lacking. In this study, we investigated, classified, and compared the hydrogenases and their expression profiles of the three alphaproteobacterial methanotrophs *Methylocystis bryophila* H2s^T^, “*Methylosinus acidophilus*” strain 29, and *Methylocapsa aurea* KYG^T^. This was followed by extensive chemostat cultivation to examine their CH_4_ and H_2_ oxidation characteristics under CH_4_‐limited conditions, simulating periods of low substrate availability that occur periodically in their natural habitats, such as bog lakes and forest soils.

### 4.1 CH_4_ Limitation Increases Hydrogenase Expression

Upon the addition of H_2_ to MIMS incubations with CH_4_‐limited *M. bryophila* H2s^T^, “*M. acidophilus*” 29, and *M. aurea* KYG^T^ cultures, immediate H_2_ oxidation was detected, which accelerated over time and with multiple H_2_ pulses. This acceleration occurred instantaneously upon H_2_ exposure and in the absence of CH_4_, suggesting re‐activation of the catalytic centres of hydrogenases rather than de novo synthesis of proteins. Pairwise comparison of the gene expression data of *M. bryophila* H2s^T^ grown under CH_4_‐limitation with and without H_2_ addition revealed that only the gene expression of the Hyd‐1d cytochrome *b* subunit was slightly upregulated when supplied with H_2_, while no significant upregulation of the large and small Hyd‐1d subunits was detected. Moreover, upon H_2_ supply to the CH_4_‐limited culture, Hyd‐1h was significantly downregulated. These findings imply that H_2_ does not promote hydrogenase expression and even suppresses Hyd‐1h transcription.

Surprisingly, for *Methylocystis* sp. strain SC2 pre‐cultivated on 20% CH_4_, a clear upregulation of Hyd‐1d proteins has been described after changing the gas composition to 2% H_2_ and 6% CH_4_, while no significant effect on both gene and protein expression of the constitutively transcribed Hyd‐1h was detected (Hakobyan et al. 2020). While Hyd‐1d is a low‐affinity hydrogenase enzyme (Mohammadi et al. 2017), Hyd‐1h is recognised as a high‐affinity H_2_‐scavenging hydrogenase capable of oxidising atmospheric H_2_ (Mohammadi et al. 2017; Schmitz et al. 2020), and it is constitutively expressed under energy‐limited conditions (Berney et al. 2014; Greening et al. 2014). This is consistent with our findings that Hyd‐1h is downregulated when *M. bryophila* H2s^T^ is cultivated without CH_4_ limitation or upon adding H_2_ to energy‐limited cultures, which also alleviates substrate limitation, as indicated by the doubling growth yield of our culture.

Interestingly, when the CH_4_‐limitation for *M. bryophila* H2s^T^ was lifted, both Hyd‐1d and Hyd‐1h, as well as the hydrogenase accessory genes, were downregulated when comparing gene expression levels to the CH_4_‐limited condition (both conditions without H_2_ supply). These findings confirm the constitutive expression of hydrogenases under CH_4_‐limited conditions and explain the detection of immediate H_2_ oxidation in the MIMS cell upon H_2_ supply. Thus, CH_4_ limitation, rather than the presence of H_2_, is the main driver of hydrogenase expression and activity.

Previous research has shown that in microorganisms encoding for both high‐affinity hydrogenases—like *M. aurea* KYG^T^—Hyd‐1h is active at even lower H_2_ concentrations than Hyd‐2a (Greening et al. 2014; Islam et al. 2020). In *Mycobacterium smegmatis* , Hyd‐1h and Hyd‐2a exhibit similar expression profiles under O_2_ and energy‐limited conditions, but Hyd‐2a is the main hydrogenase at elevated H_2_ concentrations (Berney et al. 2014; Greening et al. 2014). Surprisingly, transcription levels of Hyd‐2a in our *M. aurea* KYG^T^ CH_4_‐limited cultures with and without H_2_ addition were low, and there were no significant differences in Hyd‐2a expression between the two conditions. Hyd‐1h was expressed more highly than Hyd‐2a but, similar to *M. bryophila* H2s^T^, no upregulation of these hydrogenases was detected when H_2_ was supplied to a CH_4_‐limited culture. Thus, also for *M. aurea* KYG^T^, H_2_ is not the promoter of hydrogenase expression. CH_4_ limitation might be the main driver also in this MOB, but hydrogenase expression under CH_4_‐replete conditions was not tested here.

### 4.2 Hydrogenase Activity Is Affected by O_2_

Hyd‐1d and Hyd‐1h exhibit distinct oxygen sensitivities. Hyd‐1h is known to be highly O_2_ tolerant or even completely insensitive to O_2_ (Greening et al. 2015). However, the O_2_ sensitivity of the Hyd‐1h in *Cupriavidus necator* was evaluated only at elevated H_2_ levels, resulting in a continuously reduced catalytic centre (Schäfer et al. 2013). In fact, Schmitz et al. (2020) demonstrated that the purified Hyd‐1h from *M. fumariolicum* SolV was inhibited by oxygen in the absence of H_2_, casting doubt on its oxygen tolerance. Conversely, Hyd‐1d is believed to be partially O_2_ tolerant due to a unique proximal 6Cys[4Fe3S] cluster (Shomura et al. 2011). Still, the Hyd‐1d of *M. fumariolicum* SolV was found to be expressed only under low O_2_ concentrations (0.2% O_2_), while Hyd‐1h remained active under both low and ambient O_2_ levels (Mohammadi et al. 2017). For the alphaproteobacterial methanotrophs tested here, we observed a clear inhibition of H_2_ oxidation at O_2_ levels exceeding 2% O_2_ (Figure 2). Nonetheless, no differential expression of hydrogenase genes in *M. bryophila* H2s^T^ was detected when the culture was exposed to ambient O_2_ levels. Thus, rather than through upregulation and *de novo* protein synthesis, the observed acceleration of H_2_ oxidation rates at 1%–2% O_2_ in response to repeated H_2_ pulses of the CH_4_‐limited cultures may be attributed to the reactivation of the hydrogenases' active centres, which were inactivated due to O_2_ binding in the absence of H_2_. At elevated oxygen levels, the competition between H_2_ and O_2_ for the active centre will be intensified, thereby causing the observed reduction in maximum H_2_ oxidation rates.

### 4.3 CH_4_ Addition Accelerates H_2_ Oxidation Rates

The increase in H_2_ oxidation following CH_4_ addition to the culture indicates that the H_2_ oxidation rate is constrained by oxidised electron acceptors when CH_4_, or possibly other intermediates of CH_4_ oxidation, are not available. Upon CH_4_ addition in the MIMS cell, the methane monooxygenases will accept electrons for the concomitant reduction of O_2_ (Koo and Rosenzweig 2021). Electrons from H_2_ oxidation can flow directly to pMMO and sMMO through the quinone pool or via NADH, respectively. Additionally, in the absence of CH_4_, the cells will not be able to assimilate carbon, and consequently, a reduced ATP and NADH turnover is anticipated. Consequently, electron transfer through the respiratory chain from the hydrogenase might be reduced, probably again mainly due to a slower regeneration of electron carriers. As a result, H_2_ consumption will not reach its maximum capacity, and since the affinity constant is determined by the *V*_max_ and is not an inherent property of the enzyme, the absence of CH_4_ also causes a reduced *K*_m(app)_ value (from 55 to 35 nM). The Michaelis–Menten kinetics simulation and the simultaneous H_2_ and CH_4_ oxidation in a MIMS cell (Figure S2) showed that H_2_ oxidation rates can exceed CH_4_ oxidation rates below 1 μM of CH_4_. This concentration is in the lower limit previously reported for peatlands (Strack and Waddington 2008), an ecosystem in which *M. bryophila* H2s^T^ is originally isolated from (Belova et al. 2013). Previously reported CH_4_ concentrations in peatlands often are much higher; however, our findings underscore the importance of H_2_ as an electron donor during periods of substrate deprivation for these methanotrophs, such as during changes in water level dynamics and seasonal variations.

### 4.4 H_2_S Toxicity

The toxicity of H_2_S on (anaerobic) methanotrophy recently gained attention (Echeveste Medrano et al. 2024; Schmitz et al. 2023), and even H_2_S oxidation to elemental sulfur by the extremophilic verrucomicrobial methanotroph *M. fumariolicum* SolV was demonstrated (Schmitz et al. 2023). In the peat bogs where *M. bryophila* H2s^T^ naturally occurs, temporary upward diffusing H_2_S fluxes from anoxic sediments are expected. We have shown that CH_4_ oxidation rates of *M. bryophila* H2s^T^ were more strongly inhibited by H_2_S compared to H_2_ oxidation and methanol respiration rates. The inhibition of methanotrophy by H_2_S might be caused by its binding to metals such as copper, which is an essential cofactor not only for the terminal cytochrome *c* oxidases but also for pMMO activity (Pietri et al. 2011). Since H_2_ and methanol oxidation were less affected by H_2_S, we expected the presence of a sulfide‐insensitive terminal oxidase in strain H2s^T^, similar to *M. fumariolicum* SolV that possesses a *ba*_3_‐type terminal oxidase (Schmitz et al. 2023). Phylogenetic analysis of the terminal oxidases of *M. bryophila* H2s^T^ showed no presence of such a *ba3*‐type terminal oxidase, but the presence of multiple *aa3/caa3*‐type cytochromes, a *bo3*‐type and a *bd*‐cytochrome oxidase was detected, of which the latter is described as a sulfide‐insensitive terminal oxidase (Forte et al. 2016). The isolated *bd* oxidase of *Escherichia coli* was insensitive to sulfide up to 58 μM (Forte et al. 2016), and the presence of this gene in *M. bryophila* H2s^T^ might also be the reason for the continuation of H_2_ oxidation and thus, the generation of a proton motif force under the presence of H_2_S. Together, these results indicate that the methane monooxygenase of *M. bryophila* H2s^T^ is more sensitive to H_2_S than other proteins in its respiratory chain and that H_2_ is an important energy source when CH_4_ oxidation is inhibited by H_2_S. Interestingly, like *M. fumariolicum* SolV (Schmitz et al. 2023), the *M. bryophila* H2s^T^, “*M. acidophilus*” 29, and *M. aurea* KYG^T^ genomes all encode a sulfide:quinone reductase (SQR), giving these species the potential to oxidise the toxic and inhibitory compound H_2_S.

## 5 Conclusion

H_2_ is widely available in the environment as it can be produced by members of all three domains of life through fermentation, photoautotrophy, and N_2_ fixation. In soils and sediments, it can be provided by production in deeper anoxic layers or by diffusion from the atmosphere. The results of this study indicate that H_2_ is a significant energy source for methanotrophs, particularly under conditions of fluctuating CH_4_ and O_2_ availability and the potential presence of H_2_S, which are encountered in the natural habitats of the alphaproteobacterial methanotrophs like peat bogs and upland soil ecosystems. Therefore, the capacity to conserve energy from H_2_ oxidation is a highly advantageous, if not essential, trait for the proliferation of alphaproteobacterial methanotrophs in environments where these microorganisms encounter varying fluxes of CH_4_, H_2_, O_2_, and H_2_S.

## Author Contributions

**Ida F. Peterse:** conceptualization, investigation, writing – original draft, methodology, validation, visualisation, writing – review and editing, formal analysis, data curation. **Arjan Pol:** conceptualization, investigation, writing – original draft, methodology, validation, visualisation, writing – review and editing, formal analysis, data curation, supervision. **Geert Cremers:** methodology, validation, writing – review and editing. **Tom Berben:** methodology, visualisation, writing – review and editing. **Theo A. van Alen:** methodology, validation, writing – review and editing. **Huub J. M. Op den Camp:** conceptualization, writing – original draft, methodology, writing – review and editing, supervision, funding acquisition. **Annelies J. Veraart:** conceptualization, writing – original draft, writing – review and editing, supervision, funding acquisition. **Sebastian Lücker:** conceptualization, writing – original draft, writing – review and editing, supervision, funding acquisition.

## Ethics Statement

We have respected normal scientific and ethical practices throughout the study, as well as national and international regulations and conventions.

## Conflicts of Interest

The authors declare no conflicts of interest.

## Supporting information


**Data S1:** Supporting Information.

**Data S2:** Supporting Information.

**Data S3:** Supporting Information.

## Data Availability

The data that support the findings of this study are openly available in European Nucleotide Archive at https://www.ebi.ac.uk/ena/browser/view/PRJEB86141, reference number PRJEB86141.
