# Supplementary material for: Hydrogen Oxidation Benefits Alphaproteobacterial Methanotrophs Under Severe Methane Limitation
Source: Environ Microbiol. 2025 Aug 3;27(8):e70163. doi: 10.1111/1462-2920.70163 (PMC12319191; doi:10.1111/1462-2920.70163)
Supplement: Supplementary file 1 — Data S1: Supporting Information. [file EMI-27-e70163-s001.docx]

# Supplementary Information

Hydrogen oxidation benefits alphaproteobacterial methanotrophs under severe methane limitation

Ida F. Peterse^1,2^, Arjan Pol^1^, Geert Cremers^1^, Tom Berben^1^, Theo A. van Alen^1^, Huub J.M. Op den Camp^1^, Annelies J. Veraart^2^, Sebastian Lücker^1^*

^1^ Department of Microbiology, Radboud Institute for Biological and Environmental Sciences, Faculty of Science, Radboud University, Nijmegen, the Netherlands

^2^ Department of Ecology, Radboud Institute for Biological and Environmental Sciences, Faculty of Science, Radboud University, Nijmegen, the Netherlands

* Correspondence: [i.peterse@science.ru.nl](mailto:i.peterse@science.ru.nl) and [s.luecker@science.ru.nl](mailto:s.luecker@science.ru.nl)

# Supplementary Material and Methods

# Chemostat optimization *Methylocapsa aurea* KYG^T^

Initially, CH_4_ was supplied in the mixed gas via a sparger, but this resulted in relatively high CH_4_ concentrations in the headspace of the bioreactor, leading to biofilm formation above the liquid phase. Periodic release of the biofilm into the liquid phase caused large fluctuations in the OD_600_. Increasing the stirring speed improved the mass transfer and reduced the CH_4_ headspace concentration, but elevated the temperature because of heat production by the motorized stirrer. Therefore, we attempted to increase CH_4_ mass transfer at a reduced stirring rate (400 rpm) by supplying CH_4_ through coiled silicon tubing (7.0 m x 1.96 mm outer/1.47 mm inner diameter; Silastic®, Q7-4750 Dow-Corning Biomedical Grade) that allows direct CH_4_ diffusion into the medium. While this substantially limited biofilm growth in the headspace, biofilm started to attach to the silicon tubing. Finally, the biofilm was released, and a steady state was reached by increasing the stirring speed to 800 rpm, as apparent from the constant OD_600_.

# Supplementary Results

# Text S1. Calculation of CH_4_ concentrations in the chemostat

The outlet gas of the chemostat containing *Methylocystis bryophila* H2s^T^ biomass (D = 0.006 h^-1^) had CH_4_ concentrations of 700-800 ppm. Gas equilibrium values in the liquid were expected to be much lower, as outlet gas concentrations do not reliably reflect dissolved gas concentrations. The actual CH_4_ conversion rate was determined from the CH_4_ concentrations in both the inlet and outlet, along with the flow rate. This conversion rate was then compared with those obtained from CH_4_ kinetic experiments using membrane inlet mass spectrometry (MIMS). The corresponding CH_4_ concentration in the liquid for this conversion rate, as measured by MIMS, was 0.44 µM. At 27 °C (the chemostat temperature), this is in equilibrium with 330 ppm CH_4_ in the headspace, calculated using Henry’s law. At a growth rate of 0.0015 h^-1^, CH_4_ outlet concentrations varied between 150-180 ppm, corresponding to ~0.11 µM CH_4,_ which is in equilibrium with 80-90 ppm in the headspace.

Measured first-order kinetic constants for CH_4_ consumption in bottles (using diluted reactor samples starting at 100 ppm) yielded concentrations as low as 45-65 ppm.

# Text S2. Description of the contaminant found in the *Methylocystis bryophila* H2s^T^ chemostat

Two MAGs were assembled from the sequencing reads: one from *M. bryophila* H2s^T^ (94.1 % abundance; 99.99% completeness; 0.6% contamination) and another from a contaminant species (4.1% abundance; 79.4% completeness; 0.6% contamination). The contaminant is a new *Variovorax sp.* and, based on average nucleotide identity (ANI), is most closely related to *Variovorax* *boronicumulans* NBRC 103145 (83.7%). Furthermore, as calculated by GTDB-Tk, *V. paradoxus* NBRC 15149, *V. beijngensis* 502^T^, and *V. gossypii* DSM 100435 share 83.6%, 83.6%, and 83.1% ANI with the new *Variovorax* sp., respectively. The versatile *V. paradoxus* type strain has been found to degrade numerous xenobiotics and recalcitrant chemical compounds (Satola et al., 2013). Interestingly, some *V. paradoxus* isolates from soils can oxidize H_2_ (possibly produced by root nodules) and have been shown to promote plant growth (Maimaiti et al., 2007). However, neither the bin of our *Variovorax* contaminant nor the unbinned contigs (1.8% of all reads) contained any hydrogenases or hydrogenase accessory proteins.

Additionally, we compared catalytic [NiFe] hydrogenase subunits via BLASTP with all sequenced *Variovorax* species and in addition to *V. paradoxus* only *Variovorax* sp. OV700 encoded for hydrogenases. *Variovorax* sp. OV700, isolated from a Populus root, shares only 82.9% ANI with our contaminant. Taken together, these results indicate that H_2_ oxidation is not a widespread trait within this genus. Therefore, we assume our contaminant does not contribute to the H_2_ oxidation measured during these experiments.

The contaminant *Variovorax* sp. lacks metabolic potential for CH_4_ and H_2_ oxidation, and no key genes associated with the nitrogen or sulfur cycle were detected in the MAG. However, the pathways for organic carbon, ethanol, and acetate oxidation and fermentation were present, indicating that this *Variovorax* sp. was likely living as a heterotroph in the chemostat culture. In addition, all three urease subunits (UreABC) were detected, indicating the potential to use urea as nitrogen source.

# Text S3. Differential gene expression in *Methylocapsa aurea* KYG^T^

When grown under CH_4_ limitation with H_2_ addition, the gene cluster encoding for the electron shuttling cluster (*fixABC)* was significantly upregulated (DL86_RS15275-DL86_RS15285; LFC 1.3; Table S5). In addition, in the *pmoCAB* operon (DL86_RS13340-DL86_RS13350), only *pmoB* was significantly downregulated during H_2_ addition. Multiple genes of the formate dehydrogenase complex, catalyzing the last step in the CH_4_ oxidation pathway from formate to CO_2_, were downregulated (DL86_RS01350-DL86_RS01360; LFC 1-1.6).

Both subunits of Group 2a [NiFe] hydrogenase were very lowly expressed (TPM 9-35) during CH_4_ limitation with and without H_2_, and no differential gene expression was detected (Table S5). The expression of Group 1h [NiFe] hydrogenase subunits was higher (TPM 350-750) but was also not differentially expressed between the two conditions.

# Supplementary Figures


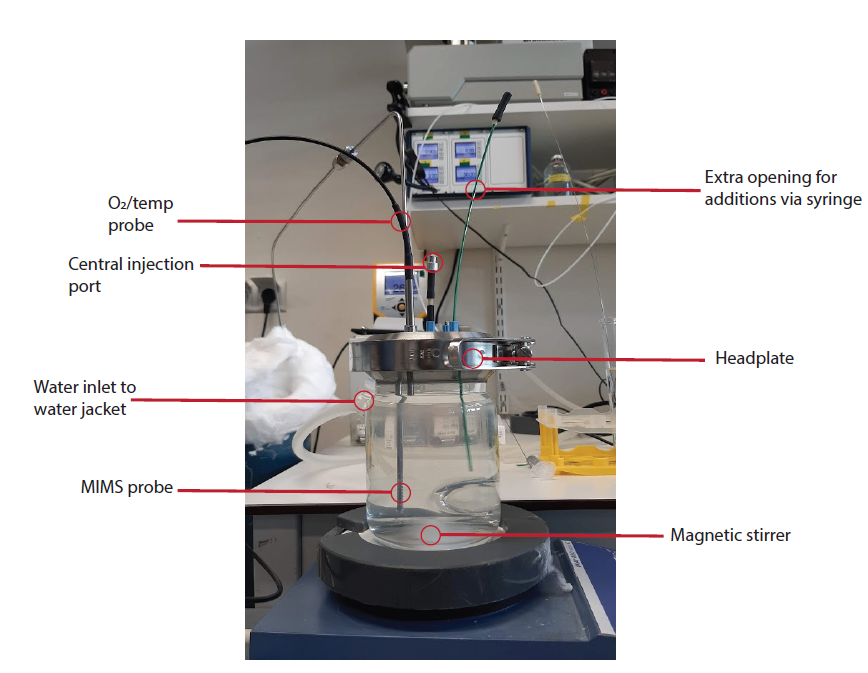


**FIGURE S1** Photograph of the membrane-inlet mass spectrometry (MIMS) setup used for kinetic experiments. The MIMS probe is enveloped by a silicon membrane and connected to the mass spectrometer via a water trap and vacuum pump. This setup allows for the real-time detection of dissolved gases in the medium within the MIMS cell.


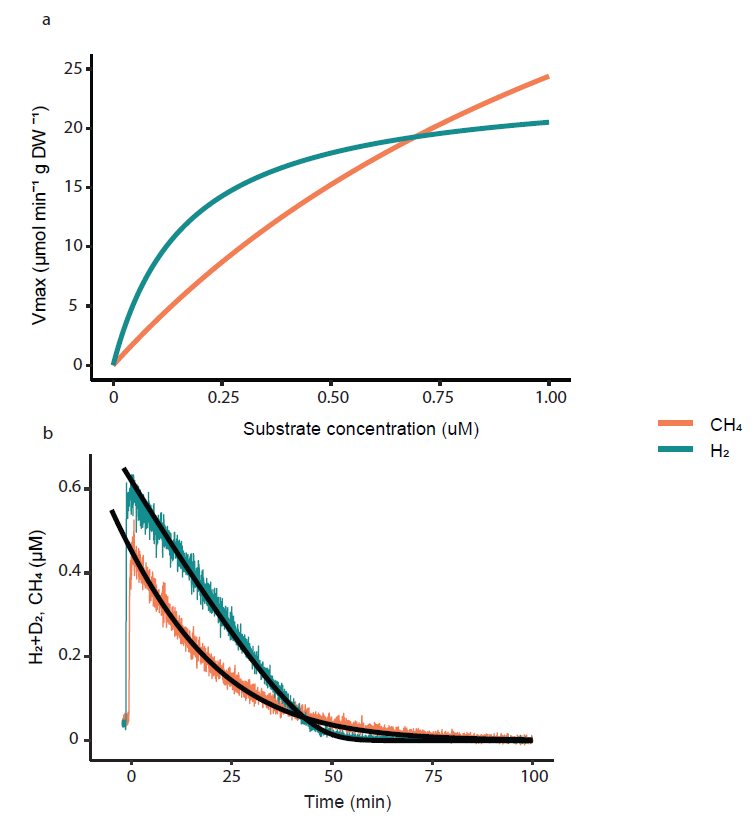


**FIGURE S2.** Simulation of Michaelis-Menten kinetics with enzymatic constants of *Methylocystis bryophila* H2s^T^. (**a**) H_2_ oxidation is faster than CH_4_ oxidation at low concentrations of CH_4_ and H_2_. This is represented by the formula V = V_max_ × ([S]/Km + [S]), where V signifies the reaction velocity and S denotes substrate concentration. The kinetic constants were derived from MIMS experiments using *M. bryophila* biomass sourced from a CH_4_-limited chemostat (D = 0.0016 h^-1^; Table 1), using V_max_ = 61 and K_S_ = 1.5 for CH_4_, and V_max_ = 24 and K_S_ = 0.17 for H_2_. (**b**) Simultaneous H_2_ and CH_4_ oxidation by *Methylocystis bryophila* H2s^T^ (D = 0.0014 h^-1^), measured in real-time in the MIMS cell, shows that H_2_ oxidation is faster than CH_4_ oxidation at concentrations below 0.5 µM.


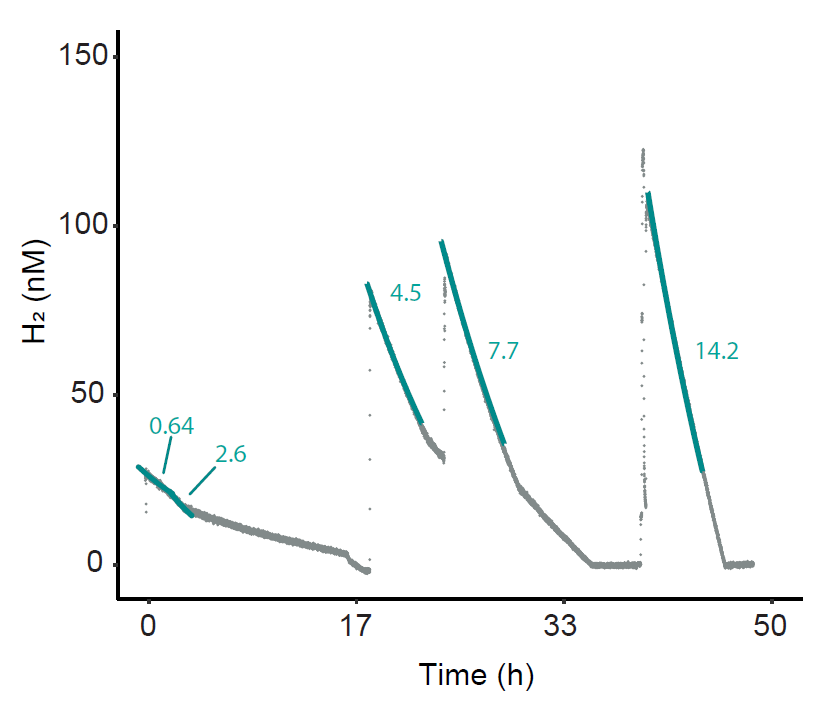


**FIGURE S3.** H_2_ oxidation by *Methylocystis* *bryophila* H2s^T^ cultivated in batch (µ = 0.065 h^-1^) before transfer to the MIMS cell. Oxidation rates accelerate over time upon the supply of H_2_ pulses. Kinetic parameters were determined by fitting simulated Michaelis-Menten kinetics through the data points (shown in blue). Rates were obtained in a single experiment and are displayed in µmol min^-1^ g^-1^ DW.


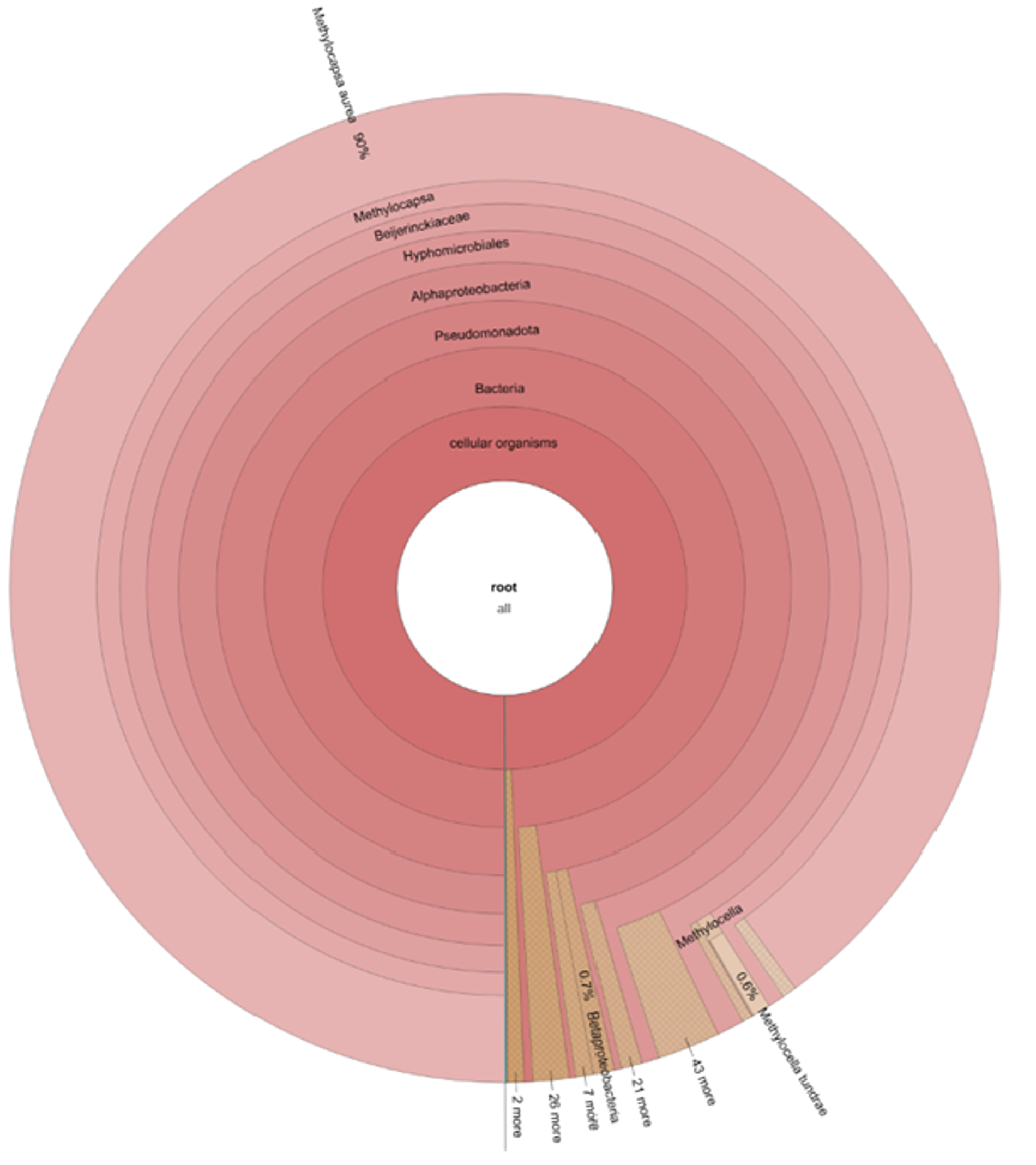


**FIGURE S4.** Krona plot representing the taxonomic classification and relative abundance of raw reads obtained with metagenomic sequencing of biomass originating from the *Methylocapsa aurea* KYG^T^ chemostat, using Kaiju.


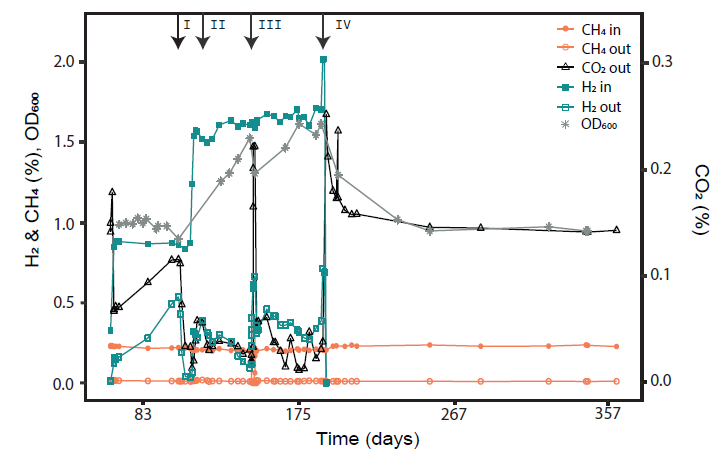


**FIGURE S5.** Continuation of Fig. 4. Long-term supply of H_2_ gas to a CH_4_- and O_2_-limited chemostat culturing *Methylocystis bryophila* H2s^T­^. CH_4_ (circles) and H_2_ (squares) concentrations in the gas inflow (filled symbols) to the bioreactor were followed, as well as the CH_4_, H_2,_ and CO_2_ (triangle) in the gas outflow (open symbols). The H_2_ concentration in the gas inflow was periodically increased and decreased. Arrow **(I)** indicates the supply of 0.5 mL 1 mM NiCl_2_ and an addition of 2 mL trace element solution, arrow **(II)** the addition of 1 mL 1 mM CoCl_2_, arrow **(III)** a two-day stop of CH_4_ supply, and arrow **(IV)** the end of H_2_ supply to the chemostat.


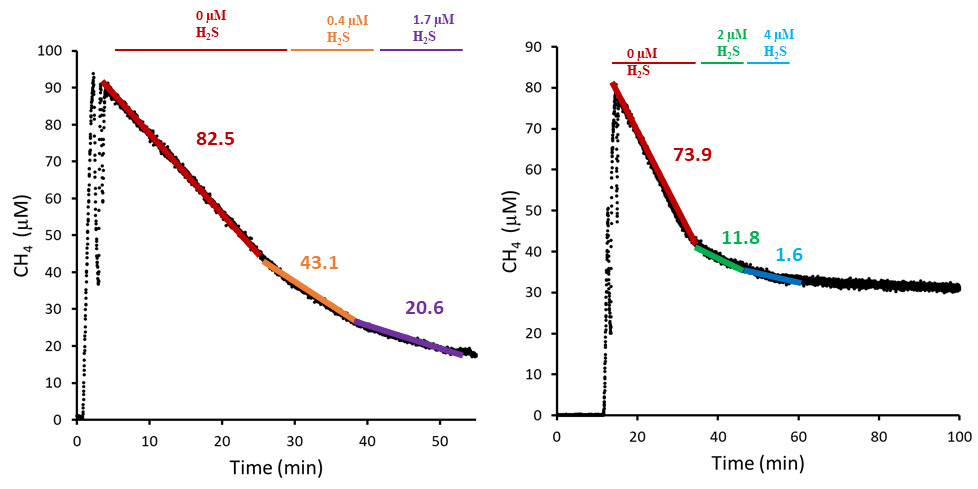


**FIGURE S6** CH_4_ oxidation rate of *Methylocystis bryophila* H2s^T^ is inhibited by H_2_S. CH_4_ consumption of cells from a CH_4_-limited chemostat culture (OD_600_ = 0.003 h^-1^) was followed in the MIMS. Different concentrations of H_2_S were added, resulting in an inhibition of CH_4_ oxidation. Kinetic parameters were determined by fitting simulated Michaelis-Menten kinetics through the data points (shown in different colors for the H_2_S concentrations indicated above the curve). Rates are displayed in µM min^-1^ g^-1^ DW. Fresh biomass was used in each experiment.


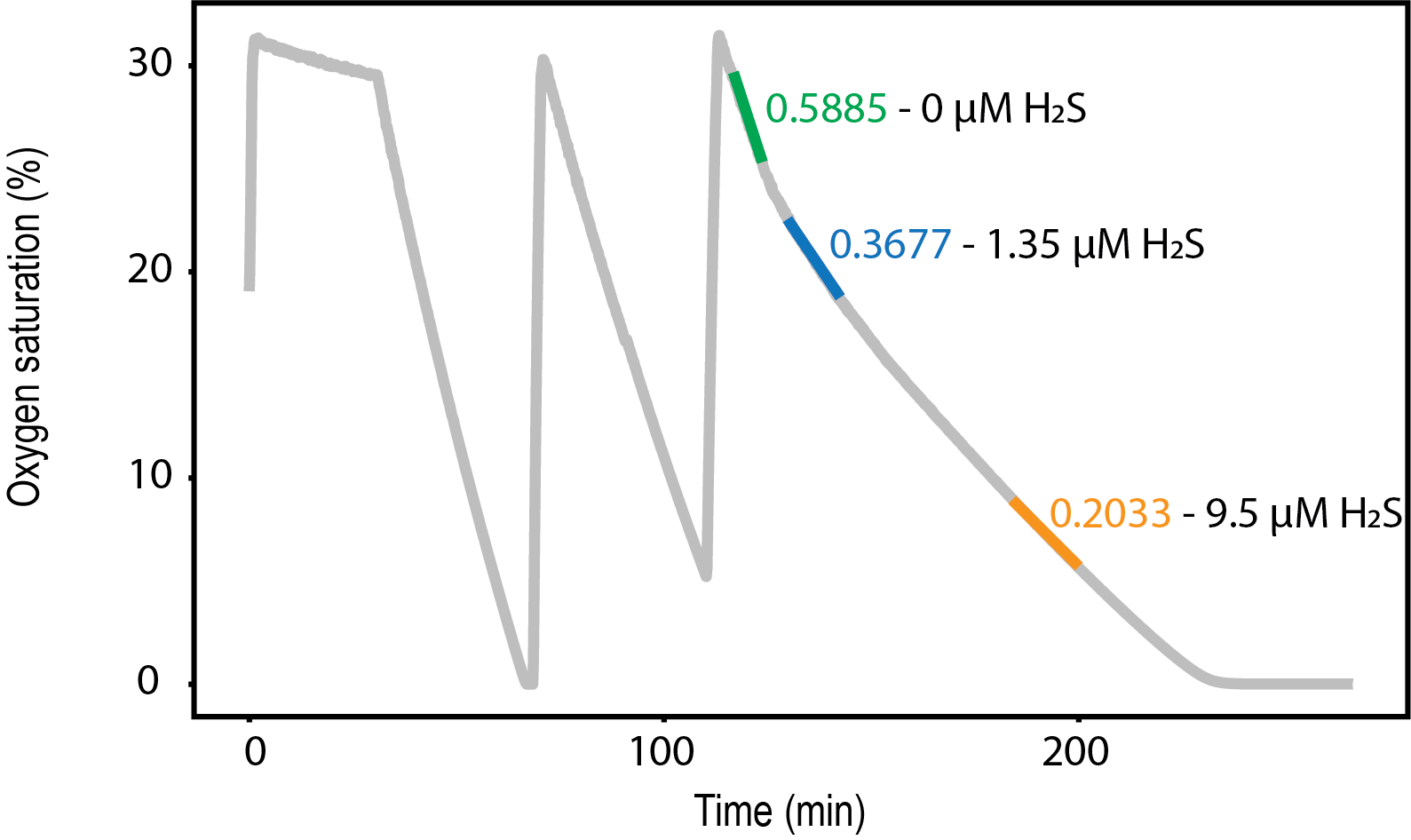


**FIGURE S7** Example of the oxygen respiration rate of *Methylocystis bryophila* H2s^T^ during increasing H_2_S inhibition of CH_3_OH oxidation measured in the MIMS cell. The incubation was initiated with 1.5 mM CH_3_OH, after which different concentrations of H_2_S were added to assess the inhibitory effect on oxygen consumption rates. Slopes of the curves were calculated by fitting a line through the data points.


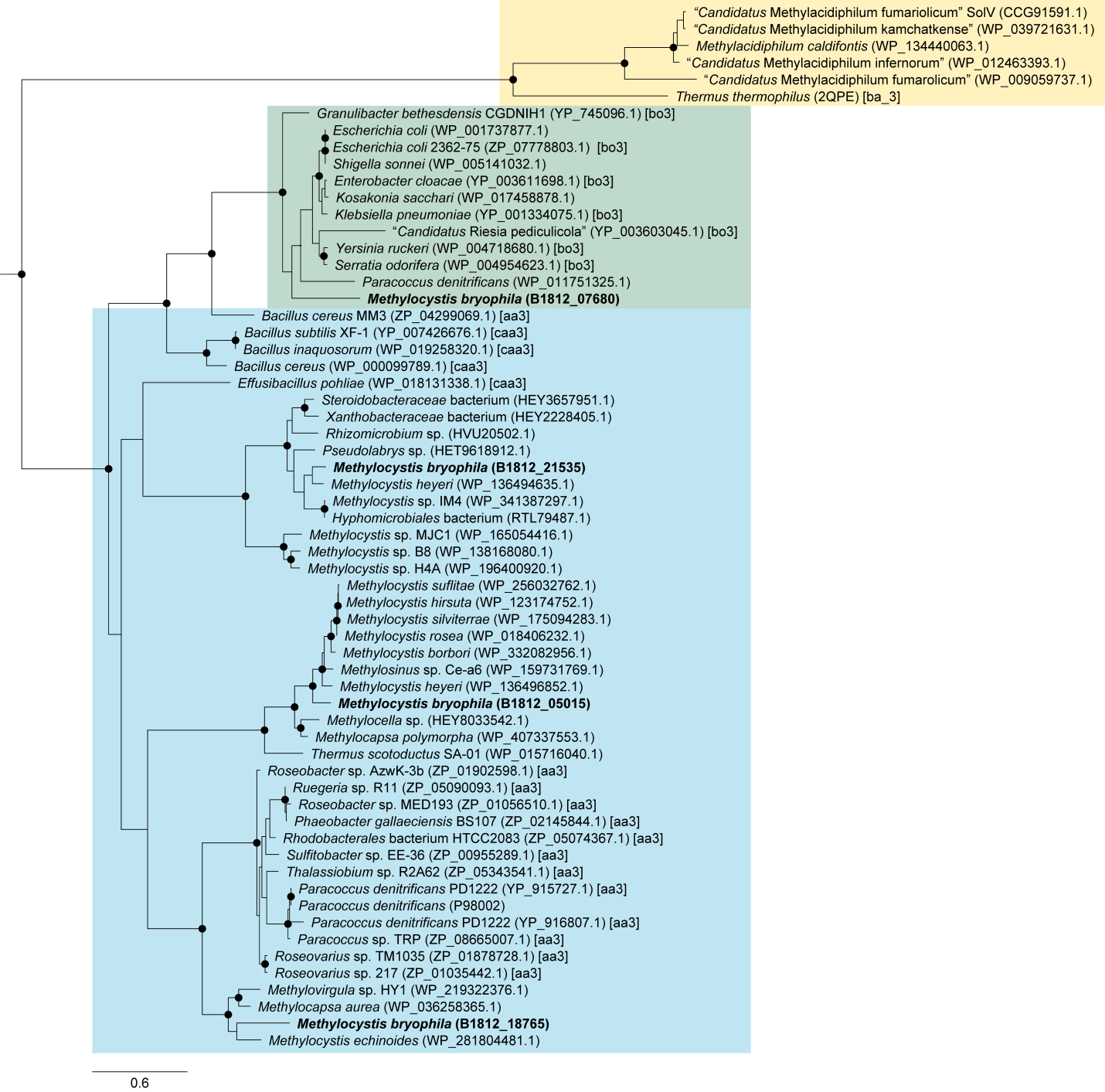


**FIGURE S8.** Maximum-likelihood tree of heme copper oxidase (HCO) protein sequences. HCOs from *Methylocystis* *bryophila* H2s^T^ are shown in bold. Yellow-shaded sequences belong to the *ba*_3_-type oxidases, green-shaded sequences to *bo*_3_-type oxidases, and blue-shaded sequences to *aa*_3_/*caa*_3_-type oxidases. Anchor sequences are annotated with their heme type in square brackets according to the classification by Bossis et al. (2014). Nodes with bootstrap support > 80% are marked with black circles. Sequences of *aa*_3_-type HCOs from *Sulfolobus* were used as the outgroup and subsequently pruned from the tree.


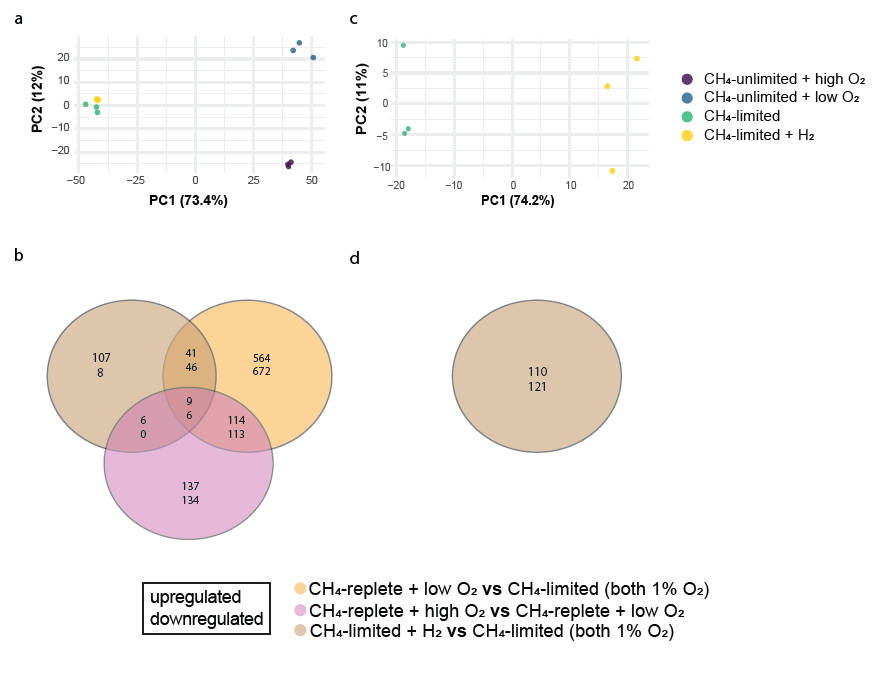


**FIGURE S9.** Principal component analysis of normalized gene expression of (**a**) *Methylocystis* *bryophila* H2s^T^ of the four conditions tested, and (**b**) *Methylocapsa aurea* KYG^T^ under two conditions. (**c, d**) Venn diagrams representing differentially expressed genes (log_2_ fold change ≥ 1 and an adjusted *p*-value ≤ 0.01) of pairwise comparisons. The upper number represents upregulated genes, and the lower number represents downregulated genes of (**c**) *M. bryophila* H2s^T^ and (**d**) *M. aurea* KYG^T^. Numbers in the overlaps indicate that genes are being up- and downregulated in multiple pairwise comparisons.


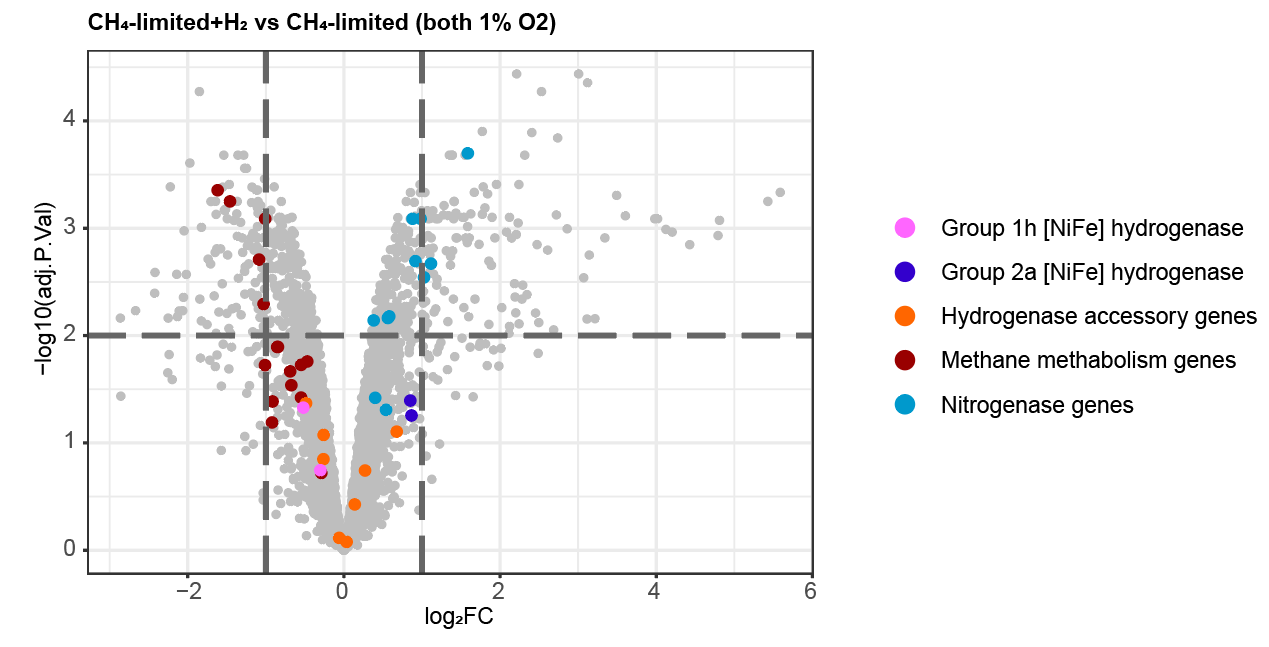


**FIGURE S10.** Volcano plot illustrating the differential gene expression of *Methylocapsa aurea* KYG^T^ between CH_4_-limited conditions with and without 1% H_2_. The x-axis represents the log_2_-fold change in transcript abundance between the first and second conditions. The y-axis depicts the negative log_10_ of the false discovery rate corrected for multiple testing. Significance thresholds are denoted by a grey dashed line, with criteria of log_2_-fold change ≥ 1 and an adjusted *p*-value of ≤ 0.01.

# Supplementary Tables

**TABLE S1** Overview of bioreactors, experiments, and analysis performed in this study. The main focus of this study was on *Methylocystis bryophila* H2s^T^. *Methylocapsa aurea* KYG^T^ and “*Methylosinus acidophilus”* 29 were used to show the conservation of H_2_ oxidation in other alphaproteobacterial methanotrophs.

|  | *Methylocystis bryophila* H2s^T^ | *Methylocapsa aurea* KYG^T^ | *“Methylosinus acidophilus”* 29 |
| --- | --- | --- | --- |
| **CH_4_-limited chemostat** | Three different dilution rates | One dilution rate | One dilution rate |
| **H_2_ oxidation activity assays (batch; Fig. 2)** | Yes, for one dilution rate | Yes | Yes |
| **H_2_ oxidation and CH_4_ oxidation kinetics (MIMS)** | Yes, for all three dilution rates (Table 1), shown for one dilution rate in Fig. 3 | Yes, data presented in Table 1 | Yes, data presented in Table 1 |
| **H_2_ and CH_4_ oxidation kinetics culture pre-grown as batch without CH_4_ limitation** | Table 1 and Fig. S3 | 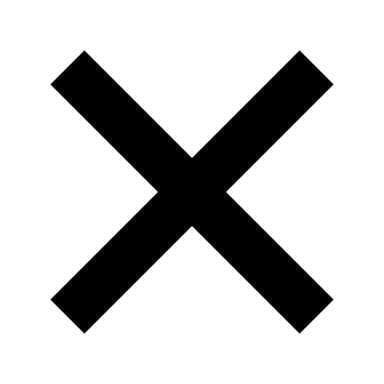 | 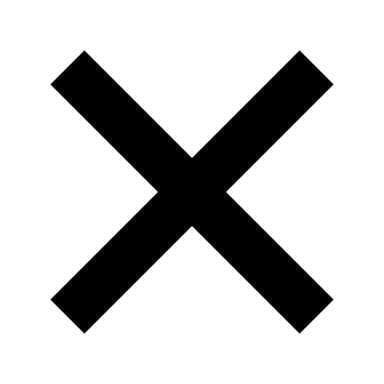 |
| **H_2_ oxidation when CH_4_ present** | Fig. 3b | 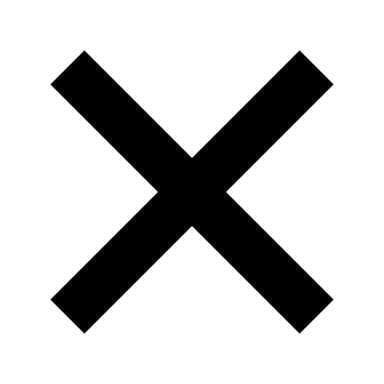 | 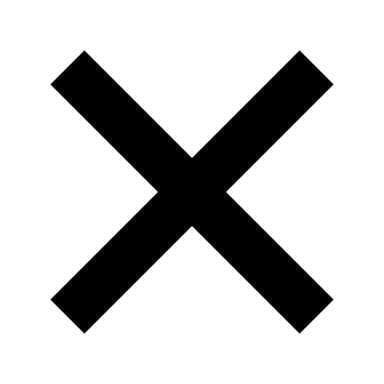 |
| **Sequencing of bioreactor cultures for purity check and metagenomic analysis (Table S3)** | Metagenome assembled and binned | Only raw read classification to demonstrate culture purity | Metagenome assembled and contigs annotated |
| **Pure culture sequencing** | 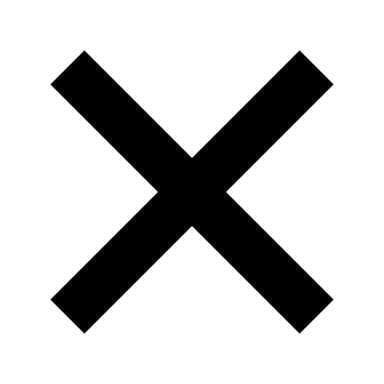 | 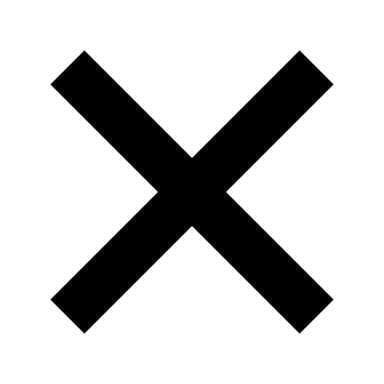 | Yes, including assembly and annotation |
| **Long-term H_2_ supply to chemostat** | Fig. 4 and Fig. S5 | Table S3 | 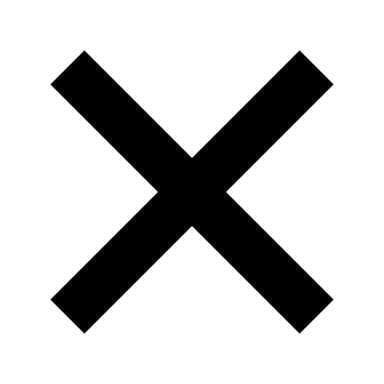 |
| **Transcriptomics** | For four conditions (Fig. 6, Fig. S9, Table S5) | For two conditions  (Fig. S10, Table S5) | 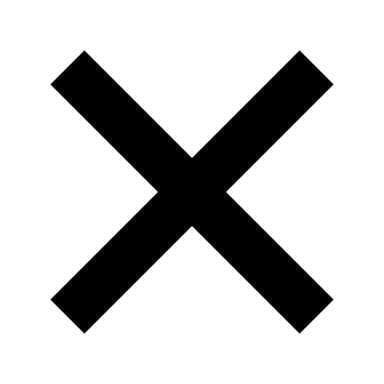 |
| **H_2_S inhibition experiments + terminal oxidase phylogenetic analysis** | Fig. 5, S6 & S7 show H_2_S inhibition data and Fig. S8 for phylogenetic analysis | 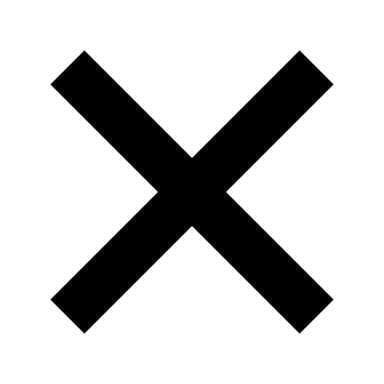 | 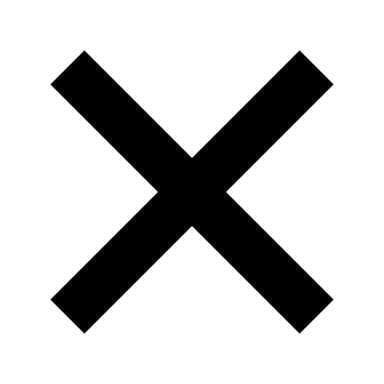 |

**TABLE S2** Overview of meta(genomic) and transcriptomic sequencing. Metagenomes of the bioreactor cultures were obtained to check the purity of the biomass. Genomic sequencing of “*Methylosinus acidophilus”* 29 was performed to obtain and annotate the genome of this strain and transcriptomic sequencing was performed to study the effect of CH_4_ limitation and H_2_ addition on the gene expression. n.d., not determined.

| **Sample & Sequencing** | **Date** | **Total and trimmed reads** | **Mapping trimmed reads to assembly (%)** | **Assembled trimmed reads in bins** | **Results** |
| --- | --- | --- | --- | --- | --- |
| *Methylocystis bryophila* H2s^T^ chemostat culture - Illumina  Metagenome-seq | June 2023 | 3,200,288 1,363,106 | 99.22 | *Variovorax* sp. bin  Size: 3.6 Mbp 4.06 % mapped reads *M. bryophila* H2s^T^ bin Size: 4.5 Mbp 94.08 % mapped reads Unbinned  Size: 0.17 Mbp 1.86 % mapped reads | No hydrogenases present in *Variovorax* sp. genome |
| *Methylocapsa aurea* KYG^T^ chemostat culture - Illumina  Metagenome-seq | June 2023 | 3,757,906 (not trim) | n.d. | n.d. | 90% of raw reads belonged to *M. aurea* KYG^T^ based on Kaiju taxonomic profiling |
| *“Methylosinus acidophilus”* 29 chemostat culture - Illumina  Metagenome-seq | Oct.2023 | 3,051,856 2,247,128 | 92.94 | n.d. | *Burkholderia* sp.  13-fold raw read coverage of contigs, containing a Group 3d [NiFe] hydrogenase *“M. acidophilus”* 29  50-fold raw read coverage of contigs |
| *“Methylosinus acidophilus*” 29  pure batch –  Illumina  & Nanopore  Genome-seq | 2022 | Illumina 5,859,384 4,302,239  Nanopore 785,250  (no trim) | 82.50 (Illumina reads against Nanopore assembly) | n.d. | **Contig length/average coverage/GC-content** Contig 0 4.3 Mbp/117.5×/0.655 Contig 1 0.32 Mbp/130.5×/0.625 Contig 2 0.23 Mbp/114.9×/0.617 Contig 3 0.21 Mbp/111.3×/0.636 Contig 4 0.056 Mbp/88.2×/0.637 Contig 5 0.031 Mbp/56.4×/0.654 |
| *Methylocystis bryophila* H2s^T^ chemostat culture –  **CH_4_-limited**  Transcriptome-seq | 2024 | 11,310,012 10,023,575* | N/A | N/A | Pseudoaligned:85.3%* |
| *Methylocystis bryophila* H2s^T^ chemostat culture –  **CH_4_-limited+H_2_**  Transcriptome-seq | 2024 | 10,205,126 9,071,381* | N/A | N/A | Pseudoaligned: 81.1%* |
| *Methylocystis bryophila* H2s^T^ chemostat culture –  **CH_4_-replete+high O_2_**  Transcriptome-seq | 2024 | 10,351,242 9,225,913* | N/A | N/A | Pseudoaligned:87.6%* |
| *Methylocystis bryophila* H2s^T^ chemostat culture –  **CH_4_-replete+low O_2_**  Transcriptome-seq | 2024 | 9,895,851 8,785,916* | N/A | N/A | Pseudoaligned:92.6%* |
| *Methylocapsa aurea* KYG^T^ chemostat culture –  **CH_4_-limited**  Transcriptome-seq | 2024 | 11,686,971 10,464,502* | N/A | N/A | Pseudoaligned: 88.5%* |
| *Methylocapsa aurea* KYG^T^ chemostat culture–  **CH_4_-limited+H_2_**  Transcriptome-seq | 2024 | 10,179,691 9,039,877* | N/A | N/A | Pseudoaligned: 89.1%* |

*All transcriptomes were sequenced in triplicates and the values in this table represent their average.

**TABLE S3** Overview of hydrogenase gene clusters and hydrogenase classification of *Methylocystis bryophila* H2s^T^, *Methylocapsa aurea* KYG^T^, and “*Methylosinus acidophilus”* 29 (provided as Supplementary Excel file)

**TABLE S4** Overview of the *Methylocapsa aurea* KYG^T^ bioreactor culture with and without H_2_ addition. Starting from 22/05/2024, H_2_ was continuously supplied to the culture, resulting in an elevated OD_600_.

| **Date** | **OD_600_** | **CH_4_ in (%)** | **CH_4_ out (%)** | **CO_2_ out (%)** | **H_2_ in (%)** | **H_2_ out (%)** |
| --- | --- | --- | --- | --- | --- | --- |
| **16/5/2024** | 0.425 | 0.0330 | 0.0046 | 0.1248 | **---** | **---** |
| **28/5/2024** | 0.52 | 0.0330 | 0.0036 | 0.1136 | 0.2134 | 0.0519 |
| **05/06/2024** | 0.57 | 0.0330 | 0.0046 | 0.0840 | 0.8078 | 0.4299 |
| **28/06/2024** | 0.88 | 0.0330 | 0.0026 | 0.1047 | 0.6620 | 0.2089 |
| **18/07/2024** | N.D. | 0.0330 | 0.0025 | 0.0861 | 0.6079 | 0.1852 |
| **26/07/2024*** | 1.08 | 0.0330 | 0.0025 | 0.0861 | 0.5951 | 0.1723 |

***** The medium influent decreased over time, affecting the OD_600_ values. We incorporated this in the yield calculations.

**TABLE S5** Overview of the differential gene expression of pairwise comparisons calculated as log_2_-fold change values and expression values calculated as transcripts per million (TPM) for *Methylocystis bryophila* H2s^T^ and *Methylocapsa aurea* KYG^T^ bioreactor cultures (provided as Supplementary Excel file).

# References

Bossis, F., De Grassi, A., Palese, L. L., & Pierri, C. L. (2014). Prediction of high-and low-affinity quinol-analogue-binding sites in the aa 3 and bo 3 terminal oxidases from Bacillus subtilis and Escherichia coli. Biochemical Journal 461 (2), 305-314.

Maimaiti, J., Zhang, Y., Yang, J., Cen, Y. P., Layzell, D. B., Peoples, M., & Dong, Z. (2007). Isolation and characterization of hydrogen‐oxidizing bacteria induced following exposure of soil to hydrogen gas and their impact on plant growth. Environmental microbiology 9 (2), 435-444.

Satola, B., Wübbeler, J. H., & Steinbüchel, A. (2013). Metabolic characteristics of the species Variovorax paradoxus. Applied microbiology and biotechnology 97, 541-560.
